# A Change in the Ion Selectivity of Ligand-Gated Ion Channels Provides a Mechanism to Switch Behavior

**DOI:** 10.1371/journal.pbio.1002238

**Published:** 2015-09-08

**Authors:** Jennifer K. Pirri, Diego Rayes, Mark J. Alkema

**Affiliations:** 1 Department of Neurobiology, University of Massachusetts Medical School, Worcester, Massachusetts, United States of America; 2 Instituto de Investigaciones Bioquímicas de Bahía Blanca, UNS-CONICET, Bahía Blanca, Argentina; Brandeis University, UNITED STATES

## Abstract

Behavioral output of neural networks depends on a delicate balance between excitatory and inhibitory synaptic connections. However, it is not known whether network formation and stability is constrained by the sign of synaptic connections between neurons within the network. Here we show that switching the sign of a synapse within a neural circuit can reverse the behavioral output. The inhibitory tyramine-gated chloride channel, LGC-55, induces head relaxation and inhibits forward locomotion during the *Caenorhabditis elegans* escape response. We switched the ion selectivity of an inhibitory LGC-55 anion channel to an excitatory LGC-55 cation channel. The engineered cation channel is properly trafficked in the native neural circuit and results in behavioral responses that are opposite to those produced by activation of the LGC-55 anion channel. Our findings indicate that switches in ion selectivity of ligand-gated ion channels (LGICs) do not affect network connectivity or stability and may provide an evolutionary and a synthetic mechanism to change behavior.

## Abstract 2

A study in *Caenorhabditis elegans* shows that switching the sign of a synapse within a neural circuit can reverse the behavioral output, providing an evolutionary and synthetic mechanism for changing behavior.

## Author Summary

Fast neurotransmission in the nervous system is mediated by ligand-gated ion channels. Within the nervous system, the sign of synaptic connections, i.e., whether they are excitatory or inhibitory, is determined by the charge of the ions that flow through these channels. In general, channels that conduct positive ions are excitatory, whereas channels that conduct negative ions are inhibitory. Here, we investigate if it is possible to flip the behavioral output of a neural circuit by changing the sign of a synapse within that circuit. The neural circuit we study controls the escape response of the nematode *Caenorhabditis elegans*. Activation of the inhibitory receptor, the tyramine-gated chloride channel LGC-55, coordinates the suppression of head movements and backward locomotion during the *C*. *elegans* escape response. We selectively mutated LGC-55 to transform it from an inhibitory into an excitatory ion channel and generated transgenic worms in which the inhibitory channel is replaced by the excitatory version of LGC-55. We show that the excitatory version of LGC-55 is properly localized to the postsynaptic compartments, is activated by the natural ligand, and does not affect network connectivity or stability. However, its expression leads to behavioral responses that are opposite to those produced by activation of the native inhibitory channel. We propose that switching the sign of a synapse not only provides a synthetic mechanism to flip behavioral output but could also be an evolutionary mechanism to change behavior.

## Introduction

**Fig 1:**
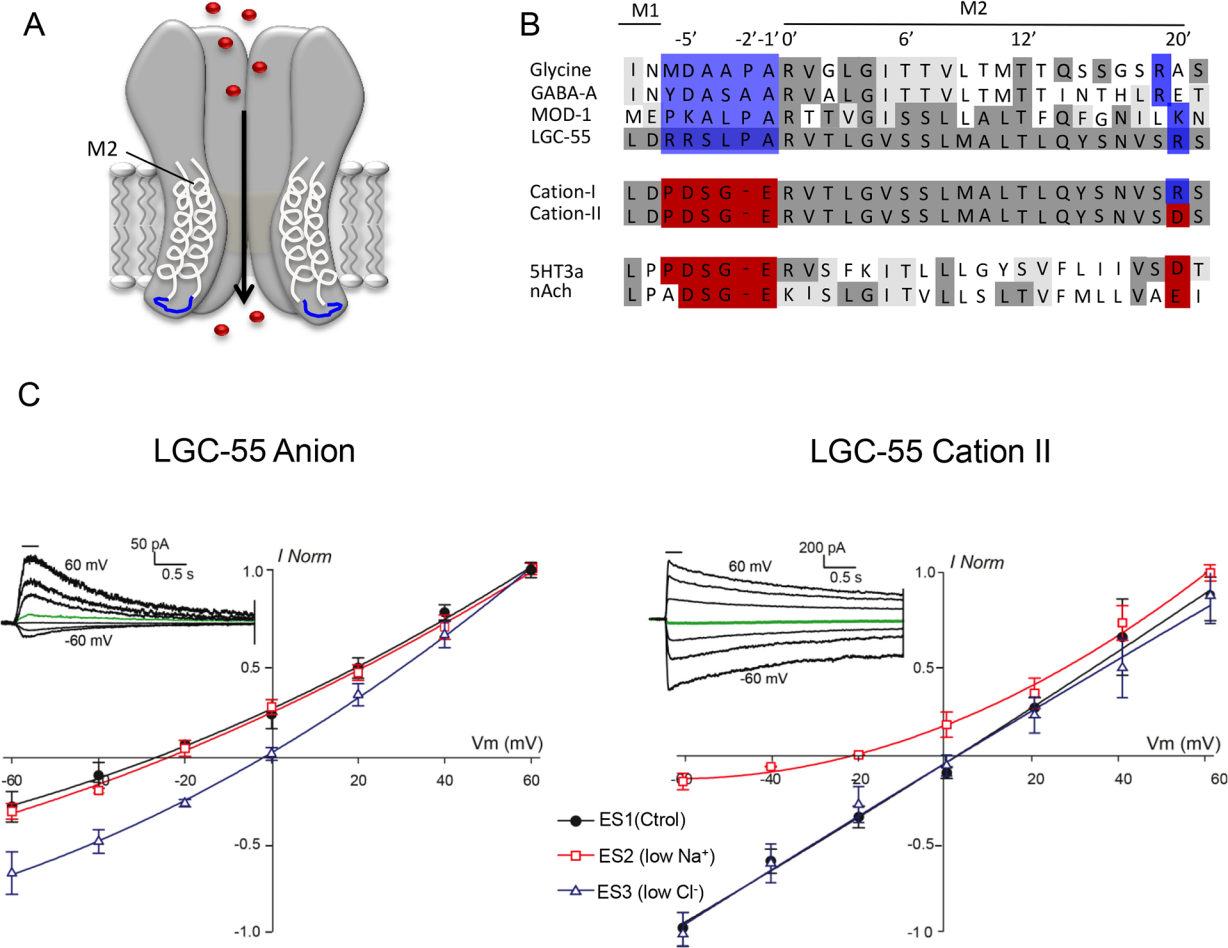
LGC-55 cation channel mutants gate sodium. (A) Cys-loop LGICs are homopentameric channels, each subunit containing four transmembrane domains. Depicted is a schematic representation of an LGIC with transmembrane domains 1 and 2 (M1, M2) in light gray. In blue is the intracellular loop that links M1 and M2, which determines the ion selectivity of the channel. (B) Alignment of M1–M2 loop region of LGC-55 with structurally related Cys-loop LGICs. Identities are shaded in dark gray, while similarities are light gray. The blue boxes indicate residues that determine selectivity of anions, while red boxes indicate those for cation selectivity. The engineered LGC-55 cation-I and cation-II channels contain the M1 loop of the cationic 5HT3a receptor. The LGC-55 cation-II channel also contains an additional mutation at the 20ʹ residue, which is predicted to enhance cation selectivity (see text for details). (C) Ion selectivity of the LGC-55 anion (left) and LGC-55 cation-II (right) receptor in cultured *C*. *elegans* muscle cells. Tyramine (TA) evoked (0.5 mM, 250 ms) currents were recorded at the holding potentials shown. Black circles: ES1 (standard solution: 150 mM Na^+^, 165 mM Cl^-^), LGC-55 anion: E_rev_ = -26.8 ± 3.1mV (*n* = 4), LGC-55 cation-II: E_rev_ = 2.4 ± 1.2 mV (*n* = 5); red squares: ES2 (low Na^+^: 15 mM Na^+^, 165 mM Cl^-^), LGC-55 anion: = -24.3 ± 1.6 mV (*n* = 4), LGC-55 cation-II: -21.9 ± 2.6 mV (*n* = 5); blue triangles: ES3 (low Cl^-^: 150 mM Na^+^, 30 mM Cl^-^), LGC-55 anion: -1.9 ± 2.3 mV (*n* = 4) LGC-55 cation-II: 1.7 ± 0.9 mV (*n* = 5). The insets show representative macrocurrents of LGC-55 anion (left) and LGC-55 cation-II (right) elicited after perfusion of 0.5 mM tyramine at membrane holding potentials ranging from -60 to +60 mV in 20 mV steps in the standard solution.

Mapping the neural connections of nervous systems is often considered to be a fundamental step in understanding behavior [1,2]. However, a neural connectivity map carries no information about the activity of neurons and the nature of the connections that each neuron makes. Neurons are embedded in neural networks, which require a delicate balance between excitation and inhibition to maintain network stability [3,4]. Homeostatic processes, conserved from invertebrates to humans, can adjust synaptic and neuronal excitability to keep neural circuits functioning within their stable dynamic range [5–8]. In these circuits, ligand-gated ion channels (LGICs) are the principal signaling components that mediate fast inhibitory and excitatory neurotransmission. The Cys-loop LGIC receptors, which include the cation-selective nicotinic acetylcholine receptors (nAChRs), serotonin type 3 receptors (5HT_3_Rs), and anion-selective GABA_A_ and glycine receptors, form pentameric complexes in the plasma membrane [9,10]. Each individual subunit contains an extracellular N-terminal domain that harbors the ligand binding domain and four transmembrane spanning domains (M1–M4) [11]. The charge selectivity of both anion and cation-selective channels is determined by residues in the M2 domain (Fig 1A and 1B). In vitro studies have shown that LGIC channels can be switched from excitatory cation-selective to inhibitory anion-selective and vice versa through substitutions in the intracellular loop between M1 and M2 [12–15]. However, it is not known whether these channels with switched ion selectivity are functional in vivo. By switching the sign of a synapse, can the behavioral output of a neural circuit be reversed, or will a switch in the sign of a synapse cause defects in network development and stability? Neuronal specification, receptor clustering, homeostatic processes, and behavioral feedback mechanisms may preclude such manipulations.

The nematode *Caenorhabditis elegans*, the only animal with a completely defined neural wiring diagram [16,17], is particularly suited to addressing these questions. The neural circuit that mediates the *C*. *elegans* escape response has been well characterized. The biogenic amine tyramine coordinates backward locomotion and the suppression of head movements during the *C*. *elegans* escape response elicited by touch to the anterior half of the body [18]. *C*. *elegans* has a single pair of tyraminergic neurons, the RIMs, which activate the homomeric tyramine-gated chloride channel, LGC-55 [19,20]. LGC-55 belongs to the Cys-loop LGIC family of receptors and is the only ionotropic tyramine receptor expressed in neurons and muscles that are directly postsynaptic to the tyraminergic neurons. Activation of LGC-55 induces the suppression of head movements and backward locomotion through the hyperpolarizaton of the neck muscles and premotor interneurons that drive forward locomotion. Here we changed the ion selectivity of LGC-55, from an inhibitory tyramine-gated anion channel to an excitatory tyramine-gated cation channel, and reintroduced the excitatory channel in the native circuit. We show that switching the sign of the synapse within the escape circuit does not affect circuit development or stability and results in opposite behavioral responses.

## Results

### Switching the Ion Selectivity of a Tyramine-Gated Ion Channel

The ion selective M2 domain of the tyramine-gated chloride channel LGC-55 is similar to the M2 domain of anionic Cys-loop receptors including the mammalian glycine receptors (GlyRs), gamma-aminobutyric acid receptors (GABA_A_Rs), and the *C*. *elegans* serotonin-gated chloride channel MOD-1 (Fig 1B). To change the ion selectivity of LGC-55, we replaced the residues of the M1–M2 loop with those that are conserved in structurally related cation channels. Using site directed mutagenesis, we generated cDNA clones encoding LGC-55 cation-I, containing the M1–M2 loop of the cationic 5HT3a channel (RRSLPA to PDSGE), and LGC-55 cation-II, which includes an additional substitution at the 20ʹ position of the M2 segment (R to D) (Fig 1B). The 20ʹ position of the M2 segment has been reported to increase the cation conductance [21,22]. To determine the ion selectivity of the engineered LGC-55 receptor, we recorded tyramine-elicited whole-cell currents in cultured muscle cells obtained from *C*. *elegans* strains that ectopically expressed either the wild type or engineered LGC-55 channel in body wall muscles. We analyzed current-voltage (I-V) relationships in varying ionic conditions: standard solution (ES1), low Na^+^ (ES2), and low Cl^-^ (ES3). The reversal potential (E_rev_) of the wild-type LGC-55 anion channel in ES1 was -26.8 ± 3.1 mV (*n* = 4) near the predicted E_rev_ for a *C*. *elegans* anion-selective channel under our conditions. A reduction of extracellular chloride concentration lead to a rightward shift of the reversal potential (E_rev_ in ES3 = -1.9 ± 2.3 mV, *n* = 4), while no significant differences in the E_rev_ values for the LGC-55 anion receptor were observed when we reduced the Na^+^ concentration (ES2), consistent with our previous findings (Fig 1C) [19]. The reversal potential of the engineered LGC-55 cation-II channel in standard solution was 2.4 ± 1.2 mV (*n* = 5), near the Goldman–Hodgkin–Katz (GHK)-predicted value for a cation-selective channel in our conditions. Reduction of the extracellular Cl^-^ concentrations did not lead to significant changes in this value (E_rev_ in ES3 = 1.7 ± 0.9 mV, *n* = 4), whereas a shift to more negative potentials is observed when we decreased the extracellular Na^+^ concentration (E_rev_ in ES2 = -21.9 ± 2.6 mV, *n* = 5).

To analyze the relative Cl^-^ and Na^+^ permeabilities (P_Cl_/P_Na_), we performed recordings using extracellular buffers containing different NaCl dilutions (1, 0.5, and 0.25 relative to the intracellular solution NaCl concentration; see Materials and Methods), and determined reversal potentials from current-voltage curves (S1 Fig). The E_rev_ values obtained for LGC-55 anion and engineered LGC-55 cation-II receptors were plotted against extracellular Cl^-^ activity (S1C Fig), and P_Cl_/P_Na_ values were obtained (see Materials and Methods [14,23]). Wild-type LGC-55 exhibited a P_Cl_/P_Na_ of 18.8, further confirming that these receptors are anion selective (S1 Fig). In contrast, the P_Cl_/P_Na_ value of the engineered LGC-55 cation-II channel was 0.19 (S1 Fig), indicating that the current passing through the chimeric LGC-55 cation-II channel is mainly carried by Na^+^ and that the Cl^-^ dependent component is negligible (Fig 1C). To further characterize the permeability properties of the LGC-55 cation-II channel, we analyzed E_rev_ shifts after altering extracellular K^+^ and Ca^2+^ concentrations (S2 Fig). An increase in the external K^+^ concentration significantly shifted the E_rev_ towards more positive membrane potentials, whereas changes in the external Ca^2+^ had no significant effects on the E_rev_ value (S2 Fig), indicating that the engineered LGC-55 is mainly permeant to monovalent cations. Our observations are consistent with previous reports showing that similar mutations in the M1–M2 linker of GlyR dramatically increase the permeability to monovalent cations but not to calcium [14].

**Fig 2:**
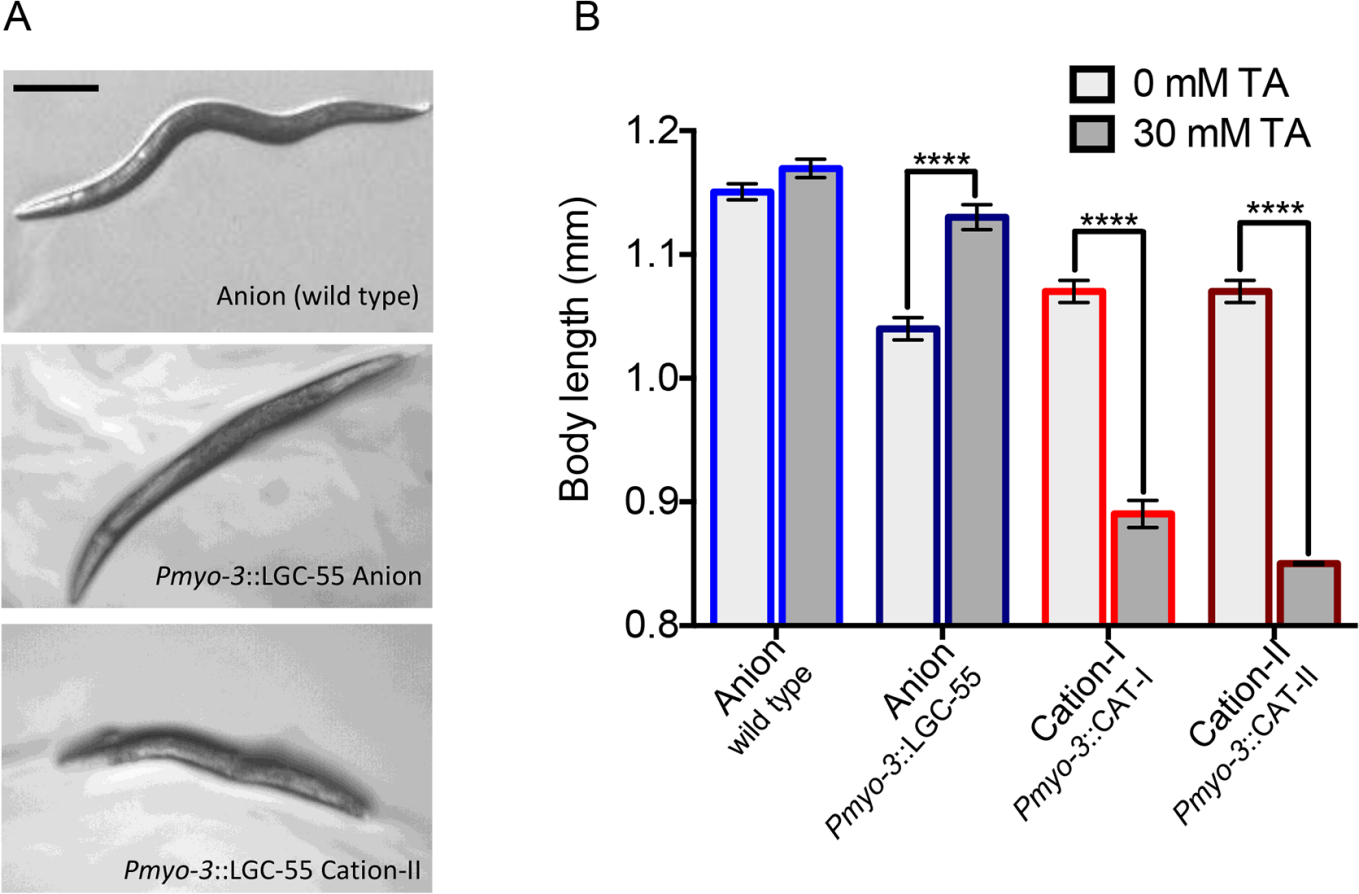
Engineered LGC-55 cation channels are functional in vivo. (A) Still images of wild-type and transgenic animals expressing LGC-55 anion or cation-II ectopically in all body wall muscle cells, on exogenous tyramine. LGC-55 anion animals paralyze in relaxed extended posture, while LGC-55 cation-II animals are hypercontracted. Scale bar = 0.25 mm. (B) Quantification of body length on exogenous tyramine (wild type, *n* = 57, P*myo-3*::LGC-55 anion(*zfEx31*), *n* = 53; P*myo-3*::LGC-55 cation-I*(zfEx120)*, *n* = 59; P*myo-3*::LGC-55 cation-II*(zfEx41)*, *n* = 55). Error bars represent the standard error of the mean (SEM). Statistical significance as indicated, *** *p* < 0.0001.

Does the engineered LGC-55 channel act as an excitatory receptor in vivo? Transgenic animals that ectopically expressed the wild-type LGC-55 anion channel in body wall muscles quickly paralyzed on plates containing exogenous tyramine. Ligand-gated chloride channels hyperpolarize the *C*. *elegans* adult body wall muscle cells, which have a low intracellular Cl^-^ concentration [24,25]. The activation of the LGC-55 anion channel caused muscle relaxation and overall body lengthening in animals overexpressing the anion channel in all body wall muscles (P*myo-3*::LGC-55 anion (*zfEx31*): Δ_body_ = 90 ± 13 μm, *n* = 53). Wild-type animals displayed a slight, although not significant (*p* = 0.07), body lengthening in response to tyramine, which could be due to the endogenous LGC-55 anion channel expression in neck muscles (wild type: Δ_body_ = 19 ± 10 μm, *n* = 57). In contrast, transgenic animals that expressed the LGC-55 cation-I or LGC-55 cation-II channel in all muscle cells became severely hypercontracted and displayed a shortened and contracted body posture in response to exogenous tyramine (P*myo-3*::LGC-55 cation-I *(zfEx120)*: Δ_body_ = -180 ± 14 μm, *n* = 59; P*myo-3*::LGC-55 cation-II *(zfEx41)*: Δ_body_ = -220 ± 33 μm, *n* = 55). Together, these data show that LGC-55 cation channels can function as excitatory receptors in vivo (Fig 2A and 2B).

### A Switch in Ion Selectivity Does Not Affect Synapse Formation

**Fig 3:**
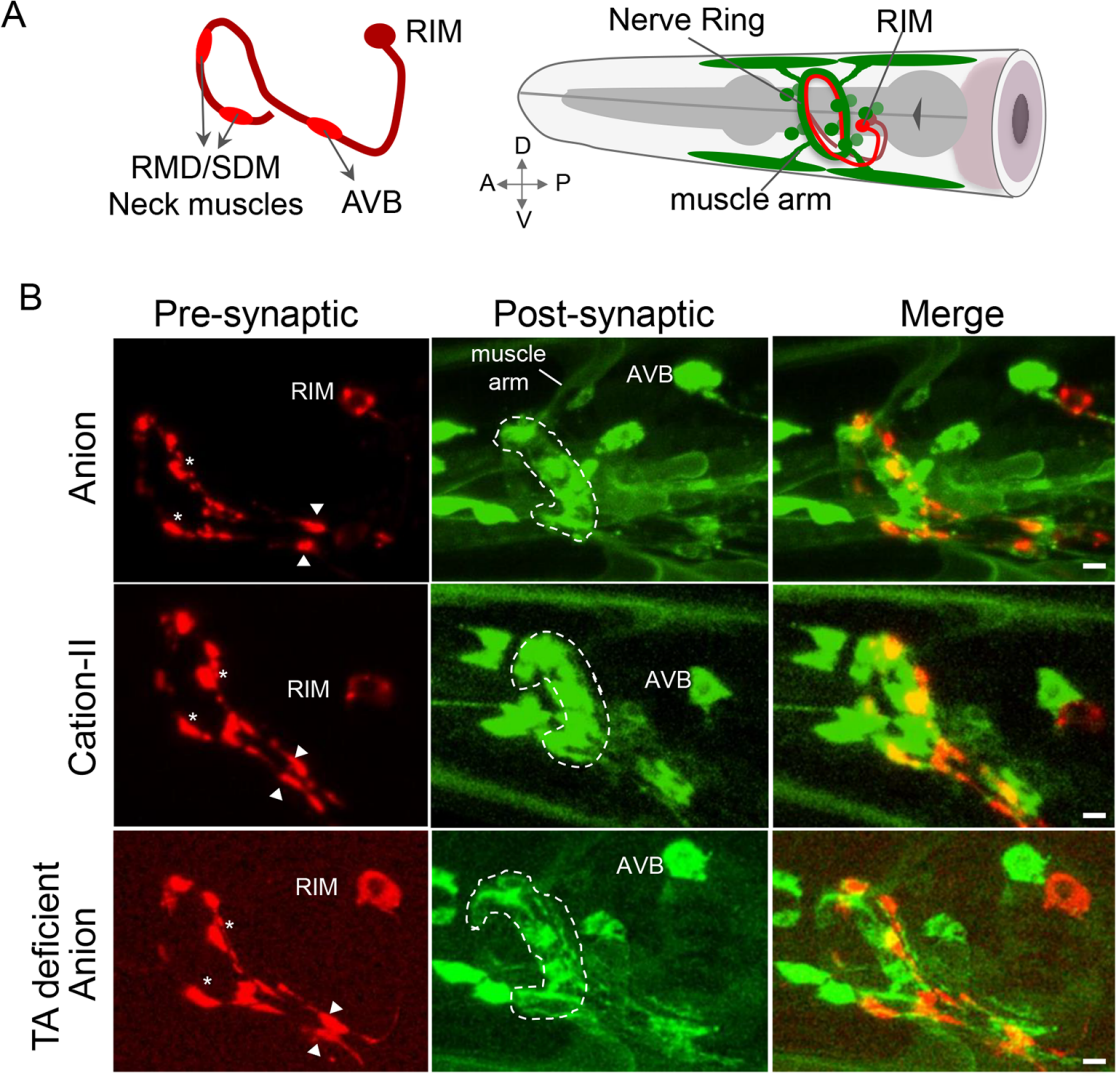
LGC-55 cation channels localize to postsynaptic specializations in the nerve ring. (A) Right: schematic diagram depicting the location of the RIM cell bodies (red) and LGC-55 expressing neurons and neck muscles in the head (green). Left: side view of the main synaptic outputs of the RIM with the AVB in its ventral process and neck muscles and the RMD/SMD motor neurons in the nerve ring. (B) Representative images of animals coexpressing the synaptic vesicle marker mCherry::RAB-3 in the tyraminergic RIM neurons (left), a translational LGC-55 anion::GFP (first row), LGC-55 cation-II::GFP reporter (center row), and a translational LGC-55 anion::GFP in tyramine-deficient, *tdc-1* mutant background (bottom row). Merge (right) shows synaptic contacts between the RIM and LGC-55 expressing neurons. The anterior is left, the nerve ring is indicated by a dashed line, and arrowheads indicate synaptic contacts between the RIM and its postsynaptic partner, the AVB neuron. Stars indicate neuromuscular junctions between the RIM and neck muscles and RMD/SMD motor neurons. The scale bar is 3 μm.

Can the LGC-55 cation channel assemble into a functional synapse? The tyraminergic RIM neuron make synaptic outputs onto the neck muscles and several head neurons that express LGC-55. To visualize tyraminergic synapses, we expressed the synaptic vesicle marker, mCherry::RAB-3 in the RIM neurons. Expression of mCherry::RAB-3 in the RIM neurons localized to axonal puncta along the ventral process and in the nerve ring, consistent with presynaptic specializations with the AVB premotor interneurons, the neuromuscular junction (NMJ) and head motor neurons, respectively (Fig 3A) [16]. To examine the localization of the tyramine-gated chloride channel, we expressed a rescuing LGC-55 anion::GFP (GFP, green fluorescent protein) translational fusion under control of the *lgc-55* promoter. LGC-55 anion::GFP receptors formed high-density clusters opposite presynaptic tyramine release sites in the nerve ring and the ventral process of the AVB premotor interneurons (Fig 3B). In transgenic animals that expressed LGC-55 cation-II::GFP, we observed clustering to synaptic specializations opposite tyramine release sites in the nerve ring and along the ventral process similar to animals expressing the LGC-55 anion channel (Fig 3B). To quantify the localization of the receptor to the post-synapse, we analyzed the pre- and post-synaptic densities of synapses from the RIM onto the AVB (S3A Fig). Both LGC-55 anion::GFP and LGC-55 cation-II::GFP cluster in discrete regions of the ventral process of the AVB ventral process opposite the tyramine release sites (S3B–S3D Fig). However, the RIM-AVB synaptic markers were slightly more diffuse in the LGC-55 cation-II transgenic animals (S3B–S3D Fig). Synaptic markers also properly localized in tyramine-deficient, *tdc-1* mutants and *lgc-55* null mutants (Fig 3B and S3 Fig). The postsynaptic densities were expanded in tyramine-deficient animals, whereas presynaptic densities were enlarged in the tyramine receptor mutants. The RIM-AVB synaptic markers were slightly more diffuse in tyramine signaling mutants and the LGC-55 cation-II transgenic animals. Our data indicate that tyramine signaling and the sign of the synapse may affect the morphology of the synapse but does not change the formation of proper pre- and postsynaptic specializations.

### A Switch in Ion Selectivity Reverses Behavior

**Fig 4:**
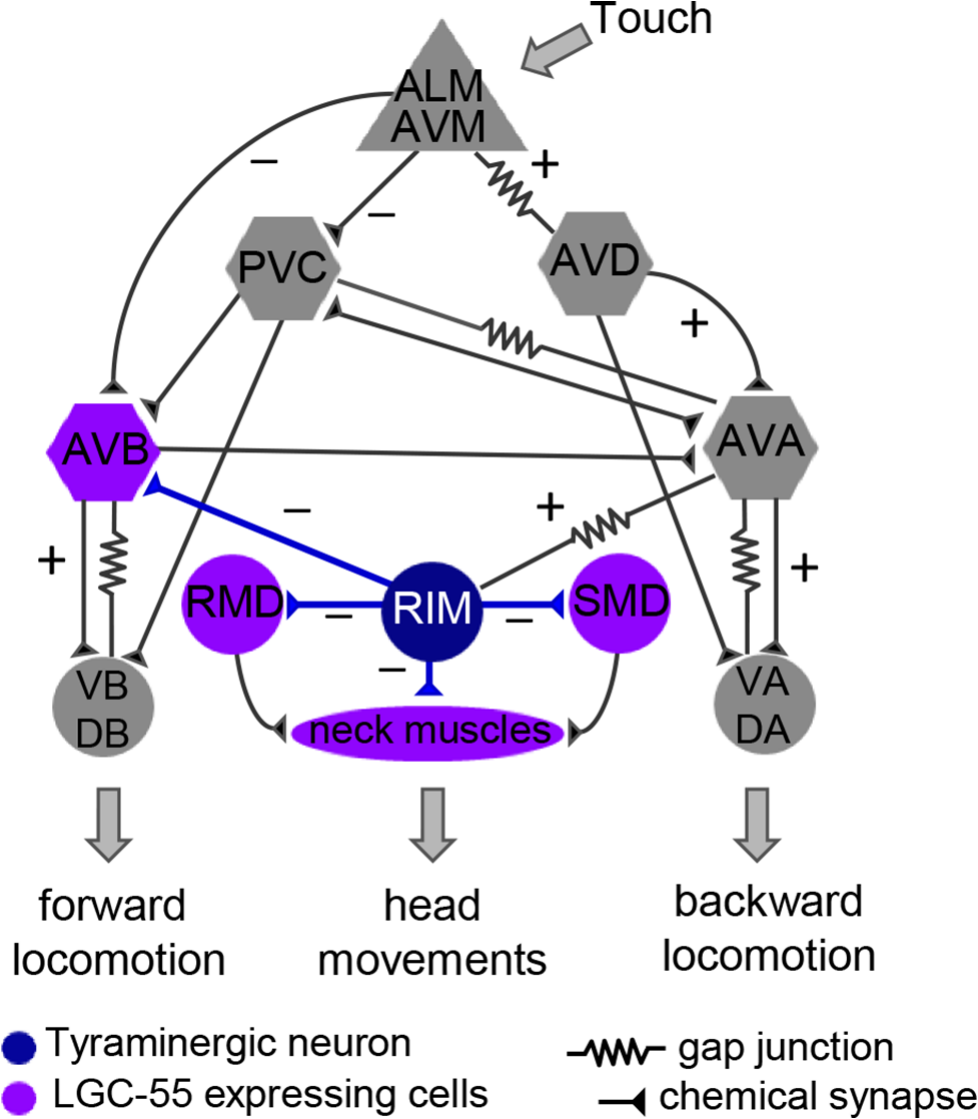
Model of the neural circuit for tyraminergic signaling in the neural escape response circuit that controls the coordination of head movements and locomotion in response to gentle anterior touch. Tyramine release from the RIM (blue) activates LGC-55 anion channel, which is expressed in the neck muscles, RMD/SMD motor neurons, and the AVB forward premotor interneuron (purple). Hyperpolarization of the neck muscles and RMD/SMD motor neurons induces neck relaxation and the suppression of head movements; hyperpolarization of the AVB forward premotor interneuron promotes backward locomotion. Tyramine signaling is induced through activation of the anterior touch sensory neurons (ALM/AVM), which activate premotor interneurons (AVD/AVA) that drive backward locomotion and are electrically coupled to the RIM (AVA-RIM). Sensory neurons are shown as triangles, premotor interneurons required for locomotion as hexagons, motor neurons as circles, and muscles as an oval.

**Fig 5:**
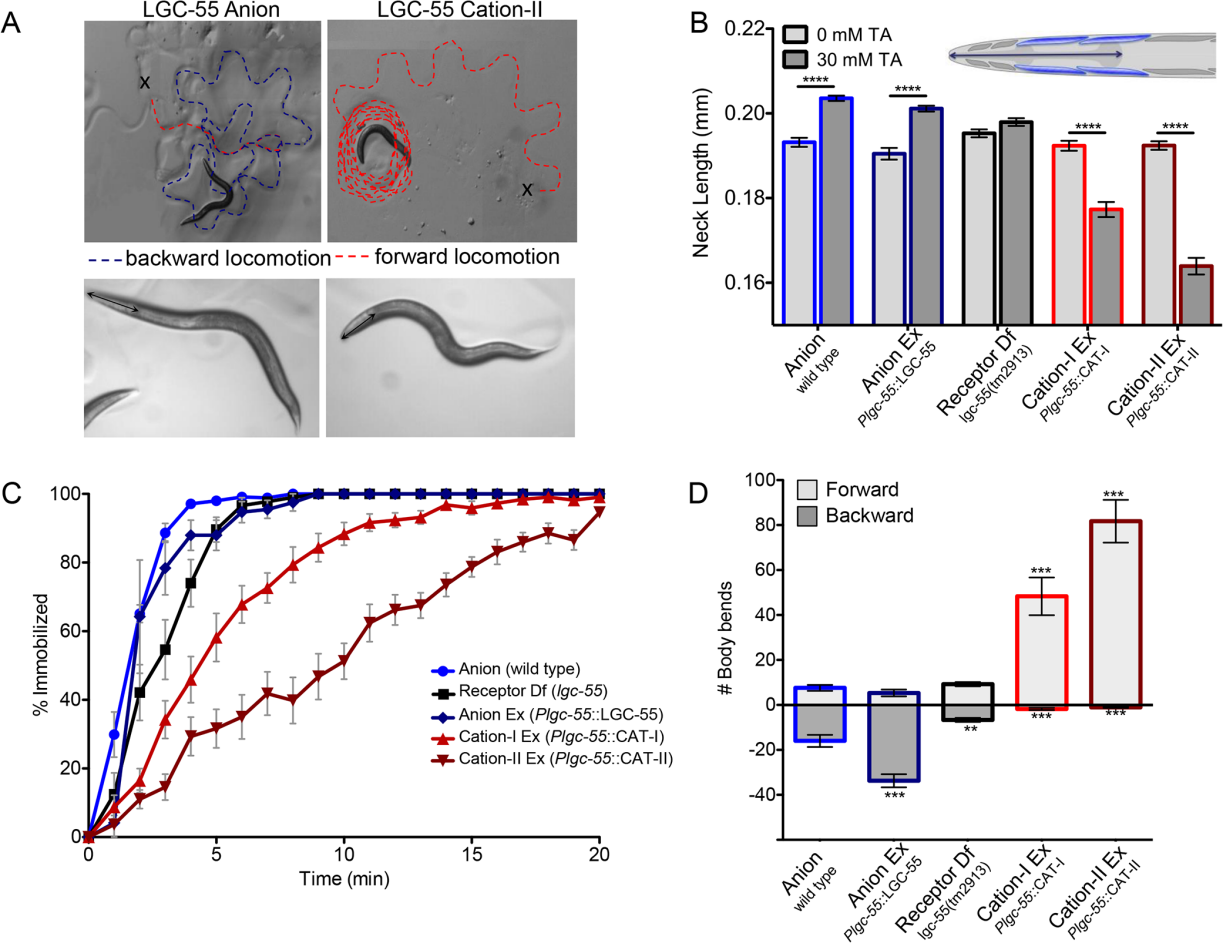
Exogenous tyramine induces long forward runs and neck contractions in animals that express the LGC-55 cation. (A) Top: still images of the locomotion pattern of transgenic animals expressing LGC-55 anion and LGC-55 cation-II prior to immobilization on 30 mM tyramine. The x marks the starting location, and the dashed red line indicates the forward locomotion, while the dashed blue line indicates backward locomotion. Bottom: still images of animals that express the LGC-55 anion or LGC-55 cation-II after five min on exogenous tyramine. The arrow-headed line indicates head length. Animals that express the LGC-55 anion exhibit a relaxation of the head muscles causing an elongation of the neck, while the expression of the LGC-55 cation channel causes contraction of the head muscles and a shortening of the neck. Scale bar, 0.2 μm. (B) LGC-55 cation transgenic animals hypercontract their neck on exogenous tyramine. Shown is the quantification of head lengths on exogenous tyramine. The length of the neck was measured from the posterior of the pharynx to the tip of the nose (inset) after 5 min on 30 mM tyramine (dark grey bars) or 0 mM tyramine (light grey bars) of wild type, *n* = 68; *Plgc-55*::LGC-55 (*lgc-55(tm2913); zfEx2*), *n* = 75; *lgc-55(tm2913)*, *n* = 65; *Plgc-55*::LGC-55 cation-I (*lgc-55(tm2913); zfEx8)*, *n* = 49; *Plgc-55*::LGC-55 cation-II (*lgc-55(tm2913)*; *zfEx40)*, *n* = 49. The error bars represent SEM. Statistical difference as indicated; *** *p* < 0.0001, two-tailed Student’s *t* test. (C) LGC-55 cation animals immobilize more slowly on exogenous tyramine. Shown is the percentage of animals immobilized by tyramine each minute for 20 min. Each data point is the mean +/- SEM for at least four trials totaling 40 or more animals. (D) LGC-55 cation animals make long forward runs on exogenous tyramine. Shown is the number of backward (dark grey bars) and forward (light grey bars) body bends made before paralysis on 30 mM tyramine of wild type, *n* = 40; *Plgc-55*::LGC-55 *(lgc-55(tm2913); zfEx2*), *n* = 29; *lgc-55(tm2913)*, *n* = 34; *Plgc-55*::LGC-55 cation-I (*lgc-55(tm2913); zfEx8)*, *n* = 28; *Plgc-55*::LGC-55 cation-II (*lgc-55(tm2913)*; *zfEx40)*, *n* = 39. Error bars represent SEM. Statistical difference from anion, ** *p* < 0.001, *** *p* < 0.0001, two-tailed Student’s *t* test.

To analyze the functional consequences of converting the ion selectivity of the LGC-55 channel, we compared the response of animals that expressed LGC-55 anion or LGC-55 cation under control of the native promoter to exogenous tyramine. LGC-55 is expressed in neck muscles, the RMD and SMD motor neurons that control foraging head movements, and the AVB premotor interneurons that drive forward locomotion (Fig 4). On plates containing exogenous tyramine, wild-type animals relax their neck and make long backward runs as a result of the activation of the LGC-55 anion receptor (S1 Movie). The animals eventually become immobilized in part through the subsequent activation of a tyramine G-protein coupled receptor SER-2 [19,26]. We have previously shown that the relaxation is mediated through hyperpolarization of the neck muscles and the cholinergic RMD and SMD head motor neurons that express LGC-55. Exogenous tyramine induced neck muscle relaxation and lengthening of the head in wild-type (LGC-55 anion) animals and *lgc-55* null mutant animals that express a rescuing LGC-55 anion transgene (wild type: Δ_head_ = 10 ± 6 μm, *n* = 68; P*lgc-55*:LGC-55(*zfEx2*): Δ_head_ = 11 ± 4 μm, *n* = 75) (Fig 5A and 5B). Head movements persisted in *lgc-55* mutants [19], with no significant change in head length (*lgc-55(tm2913)*: Δ_head_ = 3 ± 13 μm, *n* = 65). In contrast, transgenic animals that expressed the engineered LGC-55 cation-I or LGC-55 cation-II channel under control of the native promoter had a hypercontracted and shortened head length in response to exogenous tyramine (P*lgc-55*::LGC-55 cation-I (*zfEx8*): Δ_head_ = -15 ± 2 μm, *n* = 49; P*lgc-55*::LGC-55 cation-II (*zfEx40)*: Δ_head_ = -28 ± 2 μm, *n* = 49) (Fig 5A and 5B).

In wild-type animals, exogenous tyramine also induced long backward runs preceding immobilization through the LGC-55 mediated inhibition of the AVB premotor interneurons that drive forward locomotion (wild type: Δ_fwd-bwd_ = -8.46 ± 2.98 body bends, *n* = 40) (Fig 5A and 5D, S1 Movie) [19]. Backward locomotion was further increased in transgenic animals that expressed the LGC-55 anion under control of its endogenous promoter (P*lgc-55*:LGC-55(*zfEx2*): Δ_fwd-bwd_ = -28.5 ± 3.3 backward body bends, *n* = 29). *lgc-55* null mutants did not make long reversals when exposed to exogenous tyramine (*lgc-55(tm2913)*: Δ_fwd-bwd_ = 2.58 ± 0.9 body bends, *n* = 34). In sharp contrast, LGC-55 cation animals exhibit long forward runs (P*lgc-55*::LGC-55 cation-I (*zfEx8*): Δ_fwd-bwd_ = 46.5 ± 8.4 body bends, *n* = 28; P*lgc-55*::LGC-55 cation-II (*zfEx40)*: Δ_fwd-bwd_ = 80.6 ± 9.5 body bends, *n* = 39), which continued for an extended period of time (Fig 5A, 5C, and 5D; S2 Movie). The forward runs and head contractions were more pronounced in LGC-55 cation-II than in LGC-55 cation-I transgenic animals, supporting the notion that the R to D substitution at the extracellular ring of the M2 domain increases the cation conductance (Fig 5C and 5D). However, we cannot exclude the possibility that the R to D substitution may also affect gating of the engineered LGC-55 cation-II channel. Animals that express the LGC-55 anion channel become immobilized more quickly than those expressing the LGC-55 cation channel. This suggests that the immobilization on exogenous tyramine is, in part, due to the inhibition of the forward premotor interneuron, AVB (Fig 5C).

**Fig 6:**
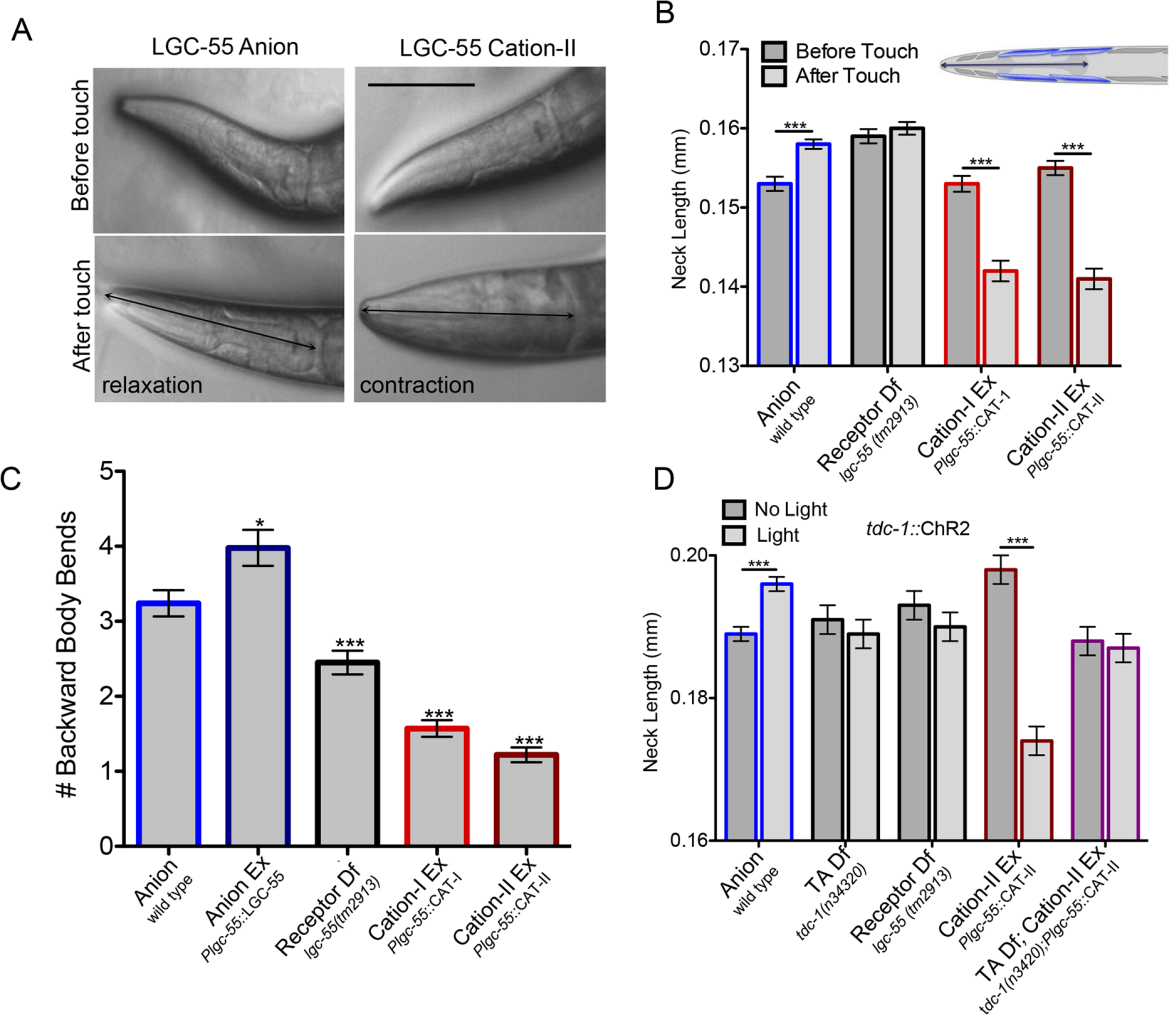
A switch in LGC-55 ion selectivity reverses behavioral output. (A) Touch induces neck relaxation in LGC-55 anion and contraction in LGC-55 cation transgenic animals. Still images of the animal’s head before (top) and after (bottom) touch stimulus. Scale bar, 0.1 mm. Arrow indicates neck length. (B) As measured in A, neck length from posterior of the pharynx to the tip of the nose before (light gray bars) and after (dark gray bars) anterior touch of wild type (*n* = 39); *lgc-55(tm2913)* (*n* = 32); *Plgc-55*::LGC-55 cation-I (*lgc-55(tm2913); zfEx8)*, *n* = 32; *Plgc-55*::LGC-55 cation-II (*lgc-55(tm2913)*; *zfEx40)*, *n* = 26. Analyses were performed in an *unc-3* mutant background to prevent backward locomotion in response to touch and to maintain the animal in the field of view at high magnification. Error bars represent SEM. Statistical difference as indicated, ** *p* < 0.001, *** *p* < 0.0001, two-tailed Student’s *t* test. (C) LGC-55 cation animals fail to execute a long reversal in response to touch. Shown is the average number of backward body bends in response to anterior touch of wild type *n* = 100; *Plgc-55*::*lgc-55 (lgc-55(tm2913); zfEx2*), *n* = 100, *lgc-55(tm2913)*, *n* = 100; *Plgc-55*:: LGC-55 cation-I (*lgc-55(tm2913); zfEx8)*, *n* = 100; *Plgc-55*::LGC-55 cation-II (*lgc-55(tm2913)*; *zfEx40)*, *n* = 100. Error bars represent SEM. Statistical difference from anion, * *p* < 0.01, *** *p* < 0.0001, two-tailed Student’s *t* test. (D) Tyramine release from the RIM activates the LGC-55 cation channel. Shown is the length of the neck before (light gray bars) and after (dark grey bars) exposure to blue light in retinal fed animals expressing the light-activated cation channel, ChannelRhodopsin 2 (ChR2), in the RIM in a wild-type background (P*tdc-1*::ChR2(*zfIs9*), *n* = 28); TA deficient (*tdc-1(n3420); Ptdc-1*::ChR2(*zfIs9*), *n* = 25); receptor deficient (*lgc-55(tm2913); Ptdc-1*::ChR2*(zfIs9)*, *n* = 28; LGC-55 cation-II (*lgc-55(tm2913);* P*lgc-55*::LGC-55 cation-II; *Ptdc-1*::ChR2 (*zfEx213)*, *n* = 20); TA deficient; LGC-55 cation-II (*tdc-1(n3420); lgc-55(tm2913);* P*lgc-55*::LGC-55 cation-II; *Ptdc-1*::ChR2(*zfEx275*), *n* = 16) animals. Analyses were performed in an *unc-3* mutant background. Blue light causes activation of the RIM and release of tyramine. Tyraminergic activation of the LGC-55 anion causes a relaxation of the neck muscles, while activation of LGC-55 cation-II causes a hypercontraction of the neck muscles. There is no response in animals that are raised on plates without all-*trans* retinal. Error bars represent SEM. Statistical difference as indicated, ** *p* < 0.001, *** *p* < 0.0001, two-tailed Student’s *t* test.

The LGC-55 anion channel was shown to coordinate backward locomotion and the suppression of foraging head movements during the *C*. *elegans* escape response elicited by gentle anterior touch [19]. To test if the LGC-55 cation channel functions in response to endogenous tyramine release, we analyzed the escape response of transgenic LGC-55 cation animals (Fig 5). Laser ablation [27], genetic analysis [18,19], and calcium imaging experiments [28,29] support the following model for the circuit that controls the escape response (Fig 4): gentle anterior touch activates the mechanosensory ALM/AVM neurons that inhibit the PVC and AVB forward premotor interneurons and activate the AVD/AVA backward premotor neurons causing the animal to move backward. The tyraminergic motor neurons (RIM) are activated during the reversal through gap junctions with the AVA backward premotor interneurons [18]. Tyramine release promotes long backward runs and induces the suppression of head movements through activation of the LGC-55 anion channel in the AVB forward premotor interneurons and neck muscles, respectively (Fig 4) [19]. In response to touch, wild-type animals suppressed head movements by relaxing their head (Δ_head_ = 5 ± 0.001 μm, *n* = 39) and reversed on average 3.14 +/- 0.18 backward body bends (*n* = 100) (Fig 6, S4 Fig, S3 Movie). *lgc-55* null mutant animals made shorter reversals than the wild type and fail to suppress the exploratory head movements during the reversal, with no significant change in head length (2.45 ± 0.15 backward body bends, *n* = 100). Strikingly, transgenic animals that expressed the LGC-55 cation channel variants contracted their neck muscles in response to touch (P*lgc-55*::LGC-55 cation-I (*zfEx8*): Δ_head_ = -11 ± 1.6 μm, *n* = 32; P*lgc-55*::LGC-55 cation-II (*zfEx40)*: Δ_head_ = -14 ± 0.009 mm, *n* = 26), and the average reversal length was markedly reduced (P*lgc-55*::LGC-55 cation-I (*zfEx8*): 1.57 ± 0.1 body bends, *n* = 100; P*lgc-55*::LGC-55 cation-II (*zfEx40)*: 1.22 ± 0.1 body bends, *n* = 100) (Fig 6A and 6C). Furthermore, transgenic LGC-55 cation animals displayed ratchety backward locomotion, often pausing during their reversal (S4 Movie). In contrast to animals expressing the LGC-55 anion, which made long spontaneous reversals, LGC-55 cation animals predominantly make short reversals, and the number of spontaneous reversals is increased (S5 Fig). We previously proposed a model in which tyramine stimulates long reversals through the LGC-55 anion mediated hyperpolarization of the AVB forward premotor interneuron [19]. Our results strongly support this model in which the substitution of the LGC-55 anion with the LGC-55 cation induces depolarization of the AVB forward premotor interneuron during the reversal and the simultaneous activation of the forward and backward locomotion programs.

We used optogenetics to determine if the contrasting behavioral responses in LGC-55 anion and LGC-55 cation animals is directly dependent on tyramine release from the RIM. Upon exposure to blue light, wild-type animals that expressed the light-activated cation channel, ChannelRhodopsin 2 (ChR2) in the RIM, relaxed their neck muscles (P*tdc-1*::ChR2(*zfIs9*): Δ_head_ = 7 ± 1.4 μm, *n* = 28) (Fig 6D, S5 Movie). In contrast, LGC-55 cation animals, which also expressed ChR2 in the RIM, hypercontracted neck muscles in response to blue-light exposure (P*lgc-55*::LGC-55 cation-II (*zfEx213)*:Δ_head_ = -24 ± 1.4 μm, *n* = 20) (Fig 5D, S6 Movie). The relaxation in the LGC-55 anion- and the contraction in the LGC-55 cation transgenic animals were abolished in tyramine-deficient *tdc-1* mutants (*tdc-1(n3420)*: Δ_head_ = -2 ± 3 μm, *n* = 25; *tdc-1(n3420);* P*lgc-55*::LGC-55 cation-II(*zfEx275*): Δ_head_ = -1 ± 3 μm, *n* = 16) (Fig 6D). These data support the notion that tyramine, released from the RIM, directly activates the tyramine-gated chloride or cation channel, LGC-55, in the postsynapstic muscle cells. Furthermore, these results indicate that the engineered LGC-55 cation channels are properly expressed and functional at the synapse within the neural circuit that modulates the *C*. *elegans* escape behavior.

## Discussion

We have shown that the replacement of the M1–M2 loop of the *C*. *elegans* inhibitory tyramine receptor, LGC-55, with that of related cation channels changes the ion selectivity from anions to monovalent cations. We have demonstrated that these engineered receptors with switched ion selectivity properly localize to the synapse and are functional in vivo. Most strikingly, we show that behavioral outputs can be reversed by switching the sign of a synapse within a neural network. Mutations in the M1–M2 linker that change the ion selectivity can also lead to changes in the gating and desensitization kinetics of the channel [30]. While the LGC-55 cation channel may also exhibit kinetic differences compared to the native LGC-55 anion channel, the opposite phenotypes observed in animals that express the LGC-55 anion channel versus those that express the LGC-55-cation channel indicate that the difference in ionic selectivity is responsible for the reversal in behavioral outputs.

**Fig 7:**
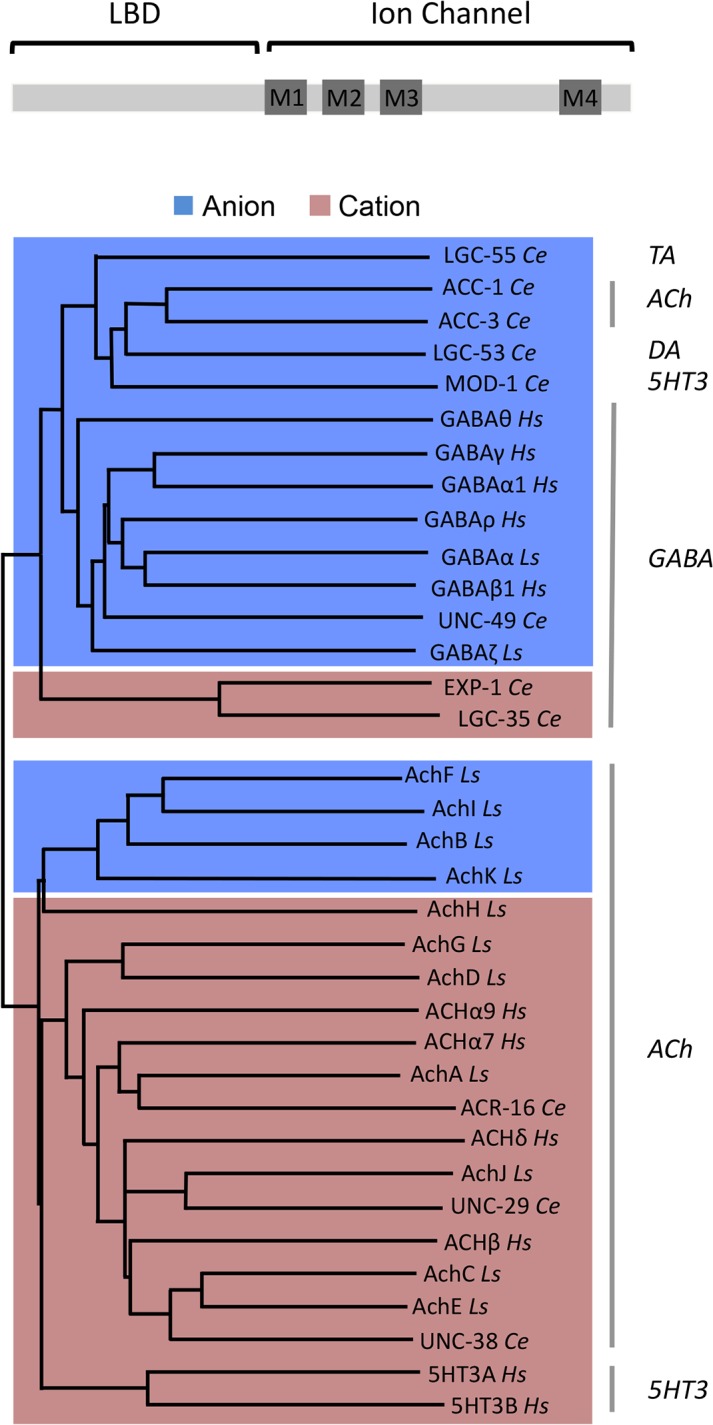
Phylogenetic comparisons of ion channel domains of members of the Cys-loop family of LGICs. LGIC phylogenetic comparison was performed on the ion channel domains of human and invertebrate LGICs. The neurotransmitter identities are indicated on the right. Blue shading indicates anionic channels, while red shading indicates cationic channels. *Ce*, *C*. *elegans*; *Ls*, *Lymnaea stagnalis*; *Hs*, *Homo sapiens*. Protein alignments were performed with ClustalW [40]. Phylogenetic analysis was performed using the neighbor joining method and midpoint rooted. Alignments and phylogenetic analyses were carried out using MacVector Software (Accelrys). GenBank accession number for the sequences used are as follows: LGC-55 *Ce*, NM_075469; ACC-1 *Ce*, NM_069314; ACC-3 *Ce*, NM_076409; LGC-53 *Ce*, NM_171813; MOD-1 *Ce*, N_741580; UNC-49 *Ce*, NM_001027610; EXP-1 *Ce*, NP_495229; LGC-35 *Ce*, NM_001027268; ACR-16 *Ce*, NM_001028676; UNC-29 *Ce*, NM_09998; UNC-38 *Ce*, NM_059071; GABAα *Ls*, X58638; GABAζ *Ls*, X71357; AchF *Ls*, DQ167349; AchI *Ls*, DQ167352; AchB *Ls*, DQ167345; AchK *Ls*, DQ167353; AchH *Ls*, DQ167351; AchG *Ls*, DQ167350; AchD *Ls*, DQ167347; AchA *Ls*, DQ167344; AchJ *Ls*, DQ167348; AchC *Ls*, DQ167344; AchE *Ls*, DQ167348; GABAθ *Hs*, NP_061028; GABAγ *Hs*, NP_775807; GABAα1 *Hs*, *NP_000797*; GABAρ *Hs*, NP_002033; GABAβ *Hs*, NP_000803; ACHα9 *Hs*, NP_060051; ACHα7 *Hs*, P36544; ACHδ *Hs*, NP_000742; ACHβ *Hs*, NP_000738; 5HT3A *Hs*, AAH04453; 5HT3B *Hs*, AAH46990.

Previous studies in both vertebrates and invertebrates have shown that neurotransmitter release is not required for the initial development of neural circuits [31,32] and does not affect clustering of postsynaptic LGICs [33]. This indicates that changing the sign of the synapse does not affect the proper wiring of neural circuits or functional synaptic transmission. While homeostatic mechanisms that maintain the balance of excitatory and inhibitory are important for network stability, these mechanisms may only occur upon perturbation within the dynamic range of the response but not when the perturbation changes the sign of the synapse. Our results indicate that neural connectivity and the sign of synaptic connections represent independent modules of the nervous system that provide a degree of freedom in generating behavioral outputs. For example, in the developing brain, GABA’s action switches from excitatory to inhibitory because of changes in the intracellular concentration of chloride [34]. Moreover, excitatory and inhibitory GABA signaling appears to coexist in the adult mammalian nervous system [35]. While in vertebrates acetylcholine (ACh) LGIC receptors are exclusively cation selective and GABA LGIC receptors are anion selective, this distinction is not as stringent in invertebrates. Molluscs have inhibitory anion-selective ACh receptors in addition to the typical excitatory cation-selective ACh LGICs [36], and *C*. *elegans* has both anion- and cation-selective ACh and GABA-gated LGICs [37–39]. Phylogenetic analysis of ion channel domains of the LGICs indicates that the *C*. *elegans* GABA-gated cation channels are more similar to the anionic GABA channels, and molluscan anion ACh channels are more closely related to the cationic ACh channels (Fig 7 and S6 Fig). This indicates that these nematode cationic GABA channels have evolved from their anionic ancestors through mutations in the ion selectivity domain, much like the engineered mutations causing the ionic switch in our engineered cation channel. The molluscan anionic ACh channels appear to have followed the opposite trajectory and changed the ion selectivity of their cationic ancestors [36]. Taken together with our results, this suggests that molecular changes in LGICs that result in a switch of the ions they flux provides an evolutionary mechanism to change behavior.

Our synaptic engineering of chemical synapses, together with the recent introduction of synthetic electrical synapses [41], indicates that the *C*. *elegans* connectome is remarkably stable. It will be interesting to see whether such manipulations are possible in neural circuits of other genetically tractable organisms. The engineering of ion selectivity of LGICs can be used as a general method to artificially change the sign of synapses in existing circuits. This synaptic engineering approach may have a broad range of applications in neuroscience, including reprogramming neurotransmitter outputs and the ability to test neural circuit models, and may present a new avenue to change behavior.

## Materials and Methods

### Strains

All *C*. *elegans* strains were grown at room temperature (22°C) on nematode growth media (NGM) agar plates with OP50 *Escherichia coli* as a food source. The strains used in this study were Bristol N2 (wild type), QW89 *lgc-55(tm2913)*, MT10661 *tdc-1(n3420)*, QW190 P*myo-3*::LGC-55 anion (*zfEx31*), QW925 P*myo-3*::LGC-55 cation-I(*zfEx120*), QW224 P*myo-3*::LGC-55 cation-II(*zfEx41*), QW606 *lgc-55*(*tm2913)*; P*cex-1*::mCherry::RAB-3; P*lgc-55*::LGC-55::GFP(*zfEx189*), QW900 *lgc-55(tm2913)*; *Pcex-1*::mCherry::RAB-3; P*lgc-55*::LGC-55 cation-II::GFP(*zfEX349*), QW1124 *tdc-1 (n3420); lgc-55(tm2913)*; P*cex-1*::mCherry::RAB-3; P*lgc-55*::LGC-55::GFP(*zfEx463*), QW827 *lgc-55(tm2913)*; *Pcex-1*::mCherry::RAB-3(*zfIs61*), QW802 *lgc-55*(*tm2913)*; P*cex-1*::mCherry::RAB-3; P*lgc-55*_short_(-120-0)::LGC-55::GFP(*zfIs72*), QW875 *lgc-55(tm2913)*; P*cex-1*::mCherry::RAB-3; P*lgc-55*_short_(-120-0)::LGC-55 cation-II::GFP(*zfIs79*), QW876 *tdc-1(n3420); lgc-55(tm2913)*; P*cex-1*::mCherry::RAB-3; P*lgc-55*_short_(-120-0)::LGC-55::GFP(*zfIs72*),QW51 *lgc-55(tm2913)*; P*lgc-55*::LGC-55(*zfEx2*), QW74 *lgc-55(tm2913);* P*lgc-55*::LGC-55 cation-I(*zfEx8*), QW219 *lgc-55*(*tm2913*); P*lgc-55*::LGC-55 cation-II(*zfEx40*), CB151 *unc-3(e151)*, QW40 *lgc-55(tm2913); unc-3(e151)*, QW538 *tdc-1(n3420); unc-3(e151)*, QW637 *lgc-55(tm2913); unc-3(e151)*; P*lgc-55*::LGC-55 cation-II(*zfEx207*), QW333 *unc-3(e151);* P*tdc-1*::ChR2(*zfIs9*), QW326 *tdc-1(n3420)*; *unc-3(e151)*; P*tdc-1*::ChR2(*zfIs9*), QW327 *lgc-55* (*tm2913*); *unc-3(e151)*; P*tdc-1*::ChR2(*zfIs9*), QW747 *tdc-1(n3420); lgc-55(tm2913); unc-3(e151)*; *tdc-1*::ChR2(*zfIs9*); P*lgc-55*::LGC-55 cation-II(*zfEx275*), and QW1283 *lgc-55(tm2913); unc-3(e151)*; *tdc-1*::ChR2(*zfIs9*); P*lgc-55*::LGC-55 cation-II(*zfEx275*)

### Molecular Biology

Standard molecular biology techniques were used. An *lgc-55* rescue construct was made by cloning an *lgc-55* genomic fragment corresponding to nucleotide (nt) -2663 to +3895 relative to the translation start site into the EcoRV site in yk1072c7 [19]. To make the chimeric LGC-55 cation-I receptor, we performed DpnI site-directed mutagenesis on the *lgc-55* rescuing construct using a primer that corresponded to the genomic sequence of the M1–M2 loop of the 5HT3a channel with 20 nt on either side homologous to the same region in LGC-55. The LGC-55 cation-II was made using DpnI site-directed mutagenesis with a primer that changed the codon at nts 1042–1044 relative to the translational start site, corresponding to a R to D substitution at the 20ʹ position of the M2 loop. LGC-55 anion::GFP and LGC-55 cation-II::GFP translational fusion constructs were made by cloning GFP into an engineered AscI restriction site in the respective P*lgc-55*::LGC-55 constructs in the sequence encoding the intracellular loop between TM3 and TM4. For muscle-specific expression of LGC-55 and LGC-55 cation-II, the full-length *lgc-55* or *lgc-55 cation-II* cDNA was cloned into pPD95.86 behind the *myo-3* promoter. For the identification of synapses in the RIM, we cloned a 1.1 kb *cex-1* promoter fragment, which drives expression in the only RIM, upstream of the mCherry::RAB-3 fusion protein from the Gateway vector, pGH8, to produce the plasmid P*cex-1*::mCherry::RAB-3. Transgenic strains were obtained by microinjection of plasmid DNA into the germline. At least three independent transgenic lines were obtained, and data are from a single representative line. Transgenic animals were generated in an *lgc-55* null background, unless otherwise noted. Transgenic animals were made by coinjecting P*lgc-55*::LGC-55 anion, P*lgc-55*::LGC-55 cation-I, P*lgc-55*::LGC-55 cation-II, P*myo-3*:LGC-55 anion, P*myo-3*::LGC-55 cation-I, P*myo-3*::LGC-55 cation-II, P*lgc-55*::LGC-55::GFP, or P*lgc-55*::LGC-55 cation-II::GFP at 20 ng/μl or P*cex-1*::mCherry::RAB-3 at 5ng/μl along with the *lin-15* rescuing plasmid pL15EK at 80 ng/μl into *lgc-55(tm2913); lin-15(n765ts)* animals, unless otherwise noted.

### Imaging

All strains were examined for colocalization of the presynaptic vesicle marker mCherry::RAB-3 in the RIM with the LGC-55 anion::GFP postsynaptic receptor using fluorescence confocal microscopy (Zeiss and Pascal imaging software). Images shown are compressed z-stacks formatted using ImageJ software.

### Isolation and Culture of *C*. *elegans* Muscle Cells

Embryonic cells were isolated and cultured as described [42]. Briefly, adult animals expressing the P*myo-3*::LGC-55 anion or LGC-55 cation-II; P*myo-3*::*GFP* transgenes were exposed to an alkaline hypochlorite solution (0.5 M NaOH and 1% NaOCl). Eggs released were treated with 1.5 U/ml chitinase (Sigma-Aldrich, St. Louis, Missouri) for 30 to 40 min at room temperature. The embryonic cells were isolated by gently pipetting and filtered through a sterile 5 μm Durapore syringe filter (Millipore Corporation, Billerica, Massachusetts) to remove undissociated embryos and newly hatched larvae. Filtered cells were plated on glass coverslips coated with peanut lectin. Cultures were maintained at RT in a humidified incubator in L-15 medium (Hyclone, Logan, Utah) containing 10% fetal bovine serum. Complete differentiation to muscle cells was observed within 24 h. Electrophysiology experiments were performed 2 to 8 d after cell isolation. Muscle cells from transgenic animals were identified by GFP expression.

### Electrophysiology

Whole-cell patch clamp recordings were performed using a HEKA EPC-9 patch clamp amplifier. Recording pipettes with a resistance of 3–7 MΩ were used. The intracellular solution (I1) contained 115 mM K-gluconate, 25 mM KCl, 0.5 mM CaCl_2_, 50 mM HEPES, 5 mM Mg-ATP, 0.5 mM Na-GTP, 0.5 mM cGMP, 0.5 mM cAMP, and 1 mM BAPTA (PH 7.4). For ionic selectivity experiments, extracellular solutions with different concentrations of Na^+^ and Cl^-^ were used: ES1 (standard solution, 150 mM NaCl, 5mM KCl, 1mM CaCl_2_, 4 mM MgCl_2_, 15 mM HEPES, 10 mM glucose, and pH 7.2 with NaOH), ES2 (low Na^+^, as ES1 except 15 mM NaCl, 135 mM NMDG-Cl) ES3 (low Cl^-^, as ES1, except 30 mM NaCl, 120 mM Na-gluconate). For K^+^ and Ca^2+^ permeability studies, the solutions used were ES4 (as ES2 except 140 mM KCl and 0 mM NMDG-Cl) and ES5 (as ES2 except 25 mM CaCl_2_, 85 mM NMDG-Cl). Current-voltage relationships were determined by measuring the current peak after 250 ms perfusion of extracellular solution containing 0.5 mM tyramine at holding potentials ranging from -60 to +60 mV in 20 mV steps.

For the dilution-potential experiments, the intracellular and extracellular buffer composition were similar to those previously reported [43]. The intracellular solution for these experiments was (I2) 145 mM NaCl, 1mM CaCl_2_, 1 mM MgCl_2,_ 1mM EGTA and 10 mM HEPES, 10 mM glucose, pH 7.2. Control extracellular solution (1NaCl, symmetrical condition) contained 145 mM NaCl, 1mM CaCl_2,_ 1 mM MgCl_2_, and 10 mM HEPES (pH 7.2). The NaCl concentration were reduced to 72.5 and 36.25 mM in the extracellular buffers used in the dilution experiments (0.5 and 0.25 NaCl, respectively). Osmolarity was maintained by adding sucrose.

P_Cl_/P_Na_ permeability ratios were obtained by fitting shifts in the E_rev_ to the GHK equation: E_rev_ = (*RT/F) ln {[P*_*Na*_ *(a*_*Na*_ *)*_*o*_ *+ (a*_*Cl*_ *)*_*i*_ *P*_*Cl*_] */* [*P*_*Na*_ *(a*_*Na*_ *)*_*i*_ *+ (a*_*Cl*_ *)*_*o*_ *P*_*Cl*_]}, where E_rev_ is the potential where the current is zero, R is the gas constant, T is the temperature, F is the Faraday’s constant, *P*_*ion*_ is the permeability of the ion, and (a_ion_) is the activity of the ion in the extracellular (subscript o) or intracellular (subscript i) solutions.

Data analyses were performed using Igor Pro software (Wavemetrics Inc, Lake Oswego, Oregon). Mean currents were fitted by a single exponential function: I_(t)_ = I_o_ exp (-t/τ_d_) + I_∞_, where I_o_ is the current at the peak, I_∞_ is the current at the end of the recording, and τ_d_ the current decay time constant. Data were normalized to I_max_, and the mean peak value in each condition was obtained after averaging three different traces (obtained not consecutively but in different voltage protocols in the same experiment). If the difference in current peak values was more than 80% for a given condition, the whole experiment was discarded. Reversal potential values are shown as mean ± standard error of 4–5 independent experiments for each extracellular solution. Curve fitting and statistical analysis were performed using Sigma Plot 11.0 (Systat Software.).

### Behavioral Assays

All behavioral analysis was performed with young adult animals (18–24 h post L4) at room temperature (22 ºC); different genotypes were scored in parallel, with the researcher blinded to the genotype. Quantification of tyramine resistance and tyramine-induced reversals was performed as described [19]. To quantify body length on exogenous tyramine, animals were placed on agar plates supplemented with 30 mM tyramine. Still frames were taken at 5 min after exposure to tyramine, and animals were measured using ImageJ software. To quantify head length on exogenous tyramine, animals were placed on 30 mM tyramine plates. Still frames were taken at 5 min after exposure to exogenous tyramine. The neck was defined as the length from the anteriormost point of the buccal cavity to the posterior of the pharyngeal bulb (as illustrated in Fig 3B). Head contraction assays in response to touch and optogenetic activation of the RIM were performed in an *unc-3(e151)* mutant background. *unc-3(e151)* animals have normal head and neck movements but have defects in the specification ventral cord neurons that affect locomotion [43]. The *unc-3(e151)* genetic background was used in these assays to prevent backward locomotion in response to touch and to maintain the animal in the field of view at high magnification that would allow for accurate neck measurement. Head lengths were measured using ImageJ software. To quantify head lengths in response to touch, animals were filmed using a Sony SX910 camera and AstroII DC software for 10 s before and after a touch posterior to the pharyngeal bulb with an eyelash. Still frames were taken from the video just prior and just after the touch. Head lengths were measured from these still frames using ImageJ software. For optogentic experiments, L4 animals were transferred to assay plates that were seeded with either OP50 *E*. *coli* that was supplemented with or without all*-trans* retinal to a final concentration of 660 μM. Animals were raised overnight on plates with or without all-*trans* retinal. To quantify head lengths in response to optogenetic activation of the RIM, animals were filmed for 10 s before and after a 2-s blue light pulse. Still frames were taken from the video just prior to and during the blue light exposure.

## Supporting Information

S1 Data Excel file containing raw data for Figs 1C, 2B, 5B–5D, and 6B–6D and S1B, S1C, S2B, S3D, S4, S5A, and S5B Figs. (XLSX)

S1 Fig P_Cl_/P_Na_ ratio of engineered LGC-55 receptor is consistent with a cation channel. (A) Representative macrocurrents of LGC-55 anion (left) and LGC-55 cation-II (right) elicited after perfusion of 0.5 mM tyramine at membrane holding potentials ranging from -40 to +40 mV in 20 mV steps using the 0.25 NaCl external solution (see below and Material and Methods). Traces in green correspond to a membrane holding potential of 0 mV. (B) Current-voltage relationships for LGC-55 anion (left) and LGC-55 cation-II (right) receptors obtained using extracellular solutions with different NaCl concentrations. 1 NaCl = NaCl 145 mM (same as intracellular solution), 0.5 NaCl = NaCl 72.5 mM, and 0.25 NaCl = NaCl 36.25 mM. (C) Plots of reversal potential shifts (Δ_rev_) against extracellular Cl^-^ activity (a_Cl_)_o_ for LGC-55 anion (left) and LGC-55 cation-II (right) receptors. The data points were fitted to the GHK equation (solid lines, see Material and Methods) to determine *P*_*Cl*_ */P*_*Na*_. The hypothetical lines for P_Cl_/ P_Na_ = ∞ or 0 are also shown (dashed lines).
(TIF)

S2 Fig LGC-55 cation channels are permeable to Na^+^ and K^+^, but not to Ca^2+^. Top: representative macrocurrents of LGC-55 cation-II elicited after perfusion of 0.5 mM TA at membrane holding potentials ranging from -60 to +60 mV in 20 mV steps in the indicated extracellular solutions. Bottom: ion selectivity of LGC-55 cation-II in cultured *C*. *elegans* muscle cells. TA-evoked (0.5 mM, 250 ms) currents were recorded at the holding potentials shown. Red squares: ES2 (low Na^+^: 15 mM Na^+^, 165 mM Cl^-^, 5 mM K^+^), LGC-55 cation-II: E_rev_ = -21.9 ± 2.6 mV (*n* = 5); purple triangles: ES4 (high K^+^: 140 mM K^+^, 1 mM Ca^2+^, 15 mM Na^+^), LGC-55 cation-II: E_rev_ = 1.9 ± 1.2 mV (*n* = 5); maroon circles: ES5 (high Ca^2+^: 5 mM K^+^, 25 mM Ca^2+^, 15 mM Na^+^), LGC-55 cation-II: E_rev_ = -20.8 ± 1.2 mV (*n* = 5).
(TIF)

S3 Fig The localization of LGC-55 cation channels to the RIM-AVB synapse. (A) Schematic diagram of the location of the synaptic outputs of the RIM onto the AVB (left: dorsal ventral view, right: side view of the head). (B) Representative images of the localization of GFP-tagged LGC-55 anion and LGC-55 cation-II opposite to presynaptic release sites from the RIM neuron (P*cex-1*::RAB-3::mCherry) of P*lgc-55*_short_::LGC-55 anion, TA-deficient (*tdc-1*), and P*lgc-55*_short_::LGC-55 cation-II::GFP animals. The presynaptic marker area indicated by the rectangle is magnified below and correlates to connections with the RIM neuron. Scale bar is 3 um. (C) Representative images of synaptic vesicle marker RAB-3::mCherry in the RIM neuron of TA-deficient (*tdc-1*), receptor-deficient (*lgc-55*), LGC-55 anion, and LGC-55 cation-II transgenic animals. The area indicated by the rectangle is magnified below and corresponds to the area of synaptic outputs of the RIM with the AVB neuron. Scale bar, 3 um. (D) Fluorescence intensity of pre- and postsynaptic densities of the RIM-AVB synapse. Fluorescence intensity at the presynapse (left) and postsynapse (right) was measured in regular intervals over 8 μm in transgenic animals expressing mCherry::RAB-3 in the RIM of wild-type (P*cex-1*::mCherry::RAB-3; P*lgc-55*::LGC-55::GFP, *n* = 19), TA-deficient (*tdc-1(n3420);* P*cex-1*::mCherry::RAB-3; P*lgc-55*::LGC-55::GFP, *n* = 14), receptor-deficient (*lgc-55(tm2913;* P*cex-1*::mCherry::RAB-3, *n* = 17), and cation-II (P*cex-1*::mCherry::RAB-3; P*lgc-55*::LGC-55 cation-II::GFP, *n* = 11) animals.
(TIF)

S4 Fig Transgenic animals that express the LGC-55 cation channel contract their heads in response to touch. Shown is the percentage of animals that contract their necks in response to touch. Positive response indicates contraction, while negative response indicates relaxation. *lgc-55* null mutants neither contract nor relax their necks, while transgenic animals expressing either LGC-55 anion or LGC-55 cation channels contract their necks in response to touch; *n* = 70 for all genotypes. See text for details. Statistical difference from LGC-55 anion, **** *p* ≤ 0.0001, two-tailed Student’s *t* test.
(TIF)

S5 Fig Transgenic animals expressing the LGC-55 cation channel have defects in spontaneous reversal behavior. (A) Number of reversals made in 3 min of wild type, 5.2 ± 0.3 body bends, *n* = 30; LGC-55 rescue, 5.2 ± 0.4 body bends, *n* = 18; *lgc-55(tm2913)*, 7.86 ± 0.5 body bends, *n* = 25; LGC-55 cation-I, 12.7 ± 1.4 body bends, *n* = 27; LGC-55 cation-II, 13.3 ± 1.9 body bends, *n* = 10. LGC-55 cation animals exhibit hyper reversal behavior. Statistical difference from LGC-55 anion. *** *p* < 0.0001, two-tailed Student’s *t* test. (B) Distribution of short (1–2 body bends) and long (3+ body bends) spontaneous reversals made in 3 min of wild type, *n* = 30; LGC-55 rescue, *n* = 18; *lgc-55(tm2913)*, *n* = 25; LGC-55 cation-I, *n* = 27; LGC-55 cation-II, *n* = 10, *p* < 0.001, two-way ANOVA. LGC-55 is expressed in the AVB forward locomotion command neuron. In wild-type animals, spontaneous release of tyramine activates LGC-55 anion, causing a hyperpolarization of the AVB leading to a long reversal. In LGC-55 cation animals, spontaneous release of tyramine causes an activation of the AVB, leading to a shortened reversal length, and an increase in the number of short reversals made in 3 min.
(TIF)

S6 Fig Alignments of the M1–M2 region of anionic and cationic LGICs. Shown is the alignment of the ion pore and M2 region of invertebrate and human LGICs used in the phylogenetic analysis in Fig 6. The neurotransmitters are indicated on the right. Identities are highlighted in grey, and blue shading indicates anionic channels, while red shading indicates cationic channels. Ce: *C*. *elegans*, Ls: *Lymnaea stagnalis*, Hs: *Homo sapiens*. Protein alignments were performed with ClustalW [40] and were carried out using MacVector Software (Accelrys). See Fig 7 for GenBank accession numbers.
(TIF)

S1 Movie Wild-type animal on agar plate containing 30 mM tyramine. Recording began immediately after the animal was placed on the plate and ended shortly after paralysis. Movie was shot at 15 frames per second (fps) and sped up five times. Wild-type animals exhibit an elongated, straightened neck and execute a long backward run before immobilization.
(MOV)

S2 Movie LGC-55 cation-II transgenic animal on a plate containing 30 mM tyramine. Recording began immediately after the animal was placed on the plate and ended shortly after paralysis. Movie was shot at 15 fps and sped up five times. Transgenic animals expressing the LGC-55 cation channel under control of the native promoter exhibit a hypercontracted neck and execute long forward runs before paralysis, behaviors opposite to that of the wild type.
(MOV)

S3 Movie Gentle anterior touch response of a wild-type animal. Wild-type animals suppress head oscillations in response to anterior touch and execute long reversals.
(MOV)

S4 Movie Gentle anterior touch response of LGC-55 cation-II transgenic animal. Animals expressing the LGC-55 cation-II channel under control of the endogenous promoter hypercontract their neck and exhibit rachety backward locomotion in response to anterior touch.
(MOV)

S5 Movie Wild-type animals expressing P*tdc-1*::ChR2 suppress head oscillations and lengthen their neck in response to blue light stimulation of tyramine release by activation of ChR2 in the RIM. (MOV)

S6 Movie LGC-55 cation-II transgenic animals expressing channelrhodopsin in the tyraminergic neurons (P*tdc-1*::ChR2) hypercontract their neck in response to blue light stimulation of tyramine release by activation of ChR2 in the RIM. (MOV)

## Abbreviations

Δ: change in length
5HT_3_R: serotonin type 3 receptor
ACh: acetylcholine
ChR2: ChannelRhodopsin-2
I-V: current-voltage
E_rev_: reversal potential
ES1: standard recording solution: 150 mM Na^+^, 165 mM Cl^-^
ES2: low Na^+^ recording solution: 15 mM Na^+^, 165 mM Cl^-^
ES3: low Cl^-^ recording solution: 150 mM Na^+^, 30 mM Cl^-^
fps: frames per second
GABA: gamma-aminobutyric acid
GABA_A_R: gamma-aminobutyric acid receptor
GFP: green fluorescent protein
GHK: Goldman–Hodgkin–Katz
GlyR: glycine receptor
LGIC: ligand-gated ion channel
M1–M4: membrane spanning domains
nACHR: nicotinic acetylcholine receptor
NGM: nematode growth media
NMJ: neuromuscular junction
nt: nucleotide
P_Cl_/P_Na_: relative Cl^-^ and Na^+^ permeability
SEM: standard error of the mean
TA: tyramine
