# Supplementary figures and images for: A Change in the Ion Selectivity of Ligand-Gated Ion Channels Provides a Mechanism to Switch Behavior
Source: PLoS Biol. 2015 Sep 8;13(9):e1002238. doi: 10.1371/journal.pbio.1002238 (PMC4562599; doi:10.1371/journal.pbio.1002238)

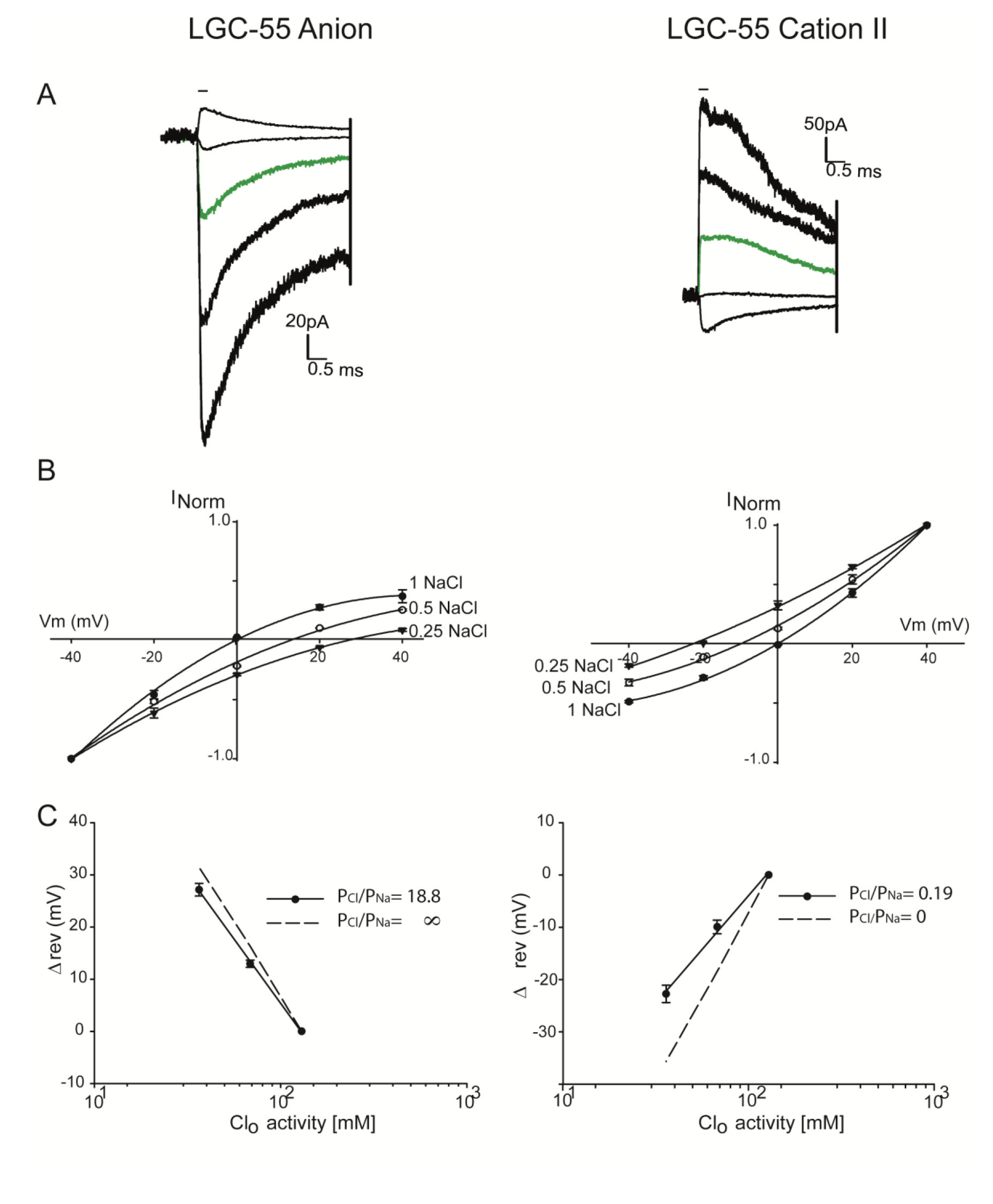

Supplement: S1 Fig — (A) Representative macrocurrents of LGC-55 anion (left) and LGC-55 cation-II (right) elicited after perfusion of 0.5 mM tyramine at membrane holding potentials ranging from -40 to +40 mV in 20 mV steps using the 0.25 NaCl external solution (see below and Material and Methods). Traces in green correspond to a membrane holding potential of 0 mV. (B) Current-voltage relationships for LGC-55 anion (left) and LGC-55 cation-II (right) receptors obtained using extracellular solutions with different NaCl concentrations. 1 NaCl = NaCl 145 mM (same as intracellular solution), 0.5 NaCl = NaCl 72.5 mM, and 0.25 NaCl = NaCl 36.25 mM. (C) Plots of reversal potential shifts (Δrev) against extracellular Cl- activity (aCl)o for LGC-55 anion (left) and LGC-55 cation-II (right) receptors. The data points were fitted to the GHK equation (solid lines, see Material and Methods) to determine P Cl /P Na. The hypothetical lines for PCl/ PNa = ∞ or 0 are also shown (dashed lines). (TIF) [file pbio.1002238.s002.tif]

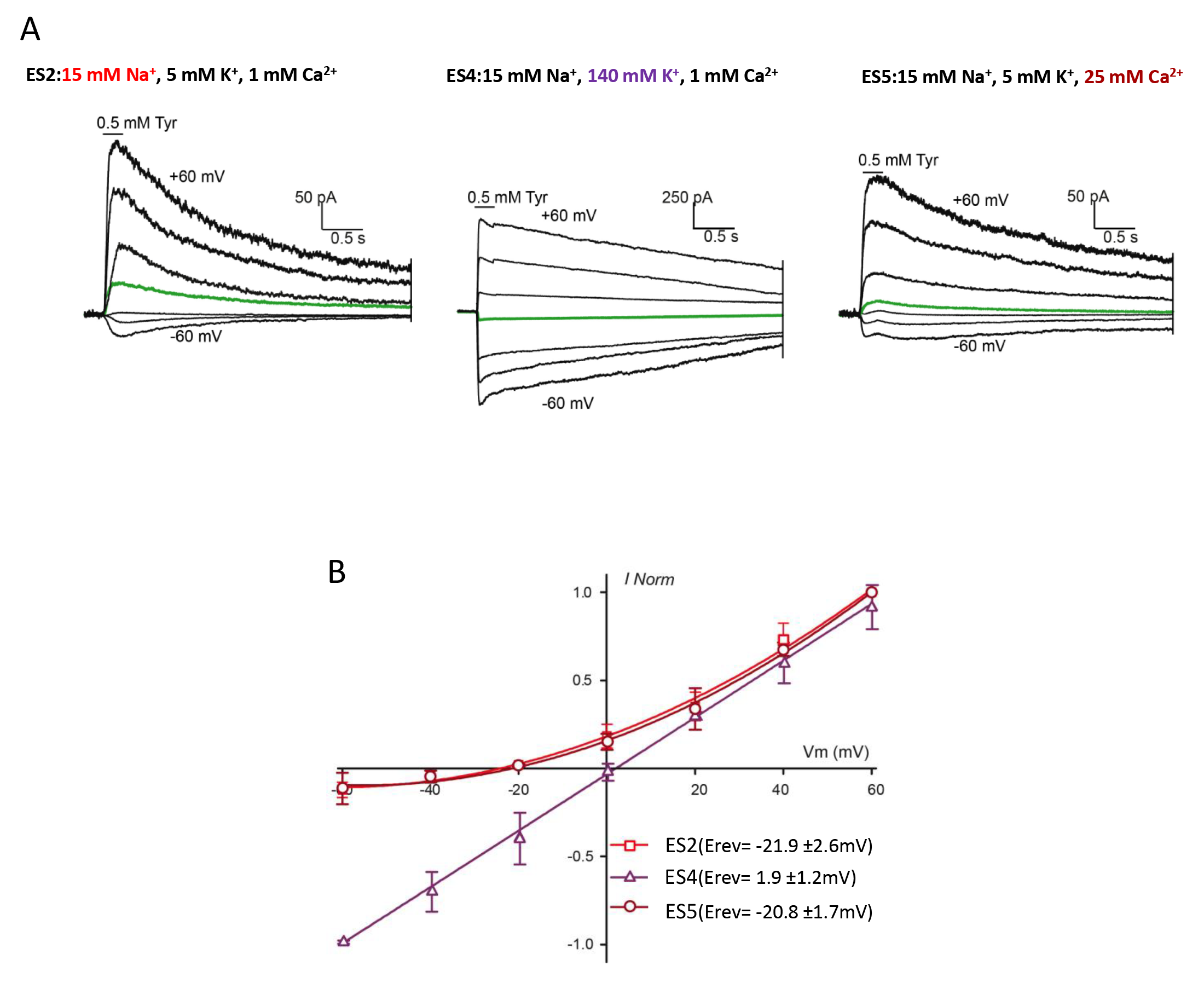

Supplement: S2 Fig — Top: representative macrocurrents of LGC-55 cation-II elicited after perfusion of 0.5 mM TA at membrane holding potentials ranging from -60 to +60 mV in 20 mV steps in the indicated extracellular solutions. Bottom: ion selectivity of LGC-55 cation-II in cultured C. elegans muscle cells. TA-evoked (0.5 mM, 250 ms) currents were recorded at the holding potentials shown. Red squares: ES2 (low Na+: 15 mM Na+, 165 mM Cl-, 5 mM K+), LGC-55 cation-II: Erev = -21.9 ± 2.6 mV (n = 5); purple triangles: ES4 (high K+: 140 mM K+, 1 mM Ca2+, 15 mM Na+), LGC-55 cation-II: Erev = 1.9 ± 1.2 mV (n = 5); maroon circles: ES5 (high Ca2+: 5 mM K+, 25 mM Ca2+, 15 mM Na+), LGC-55 cation-II: Erev = -20.8 ± 1.2 mV (n = 5). (TIF) [file pbio.1002238.s003.tif]

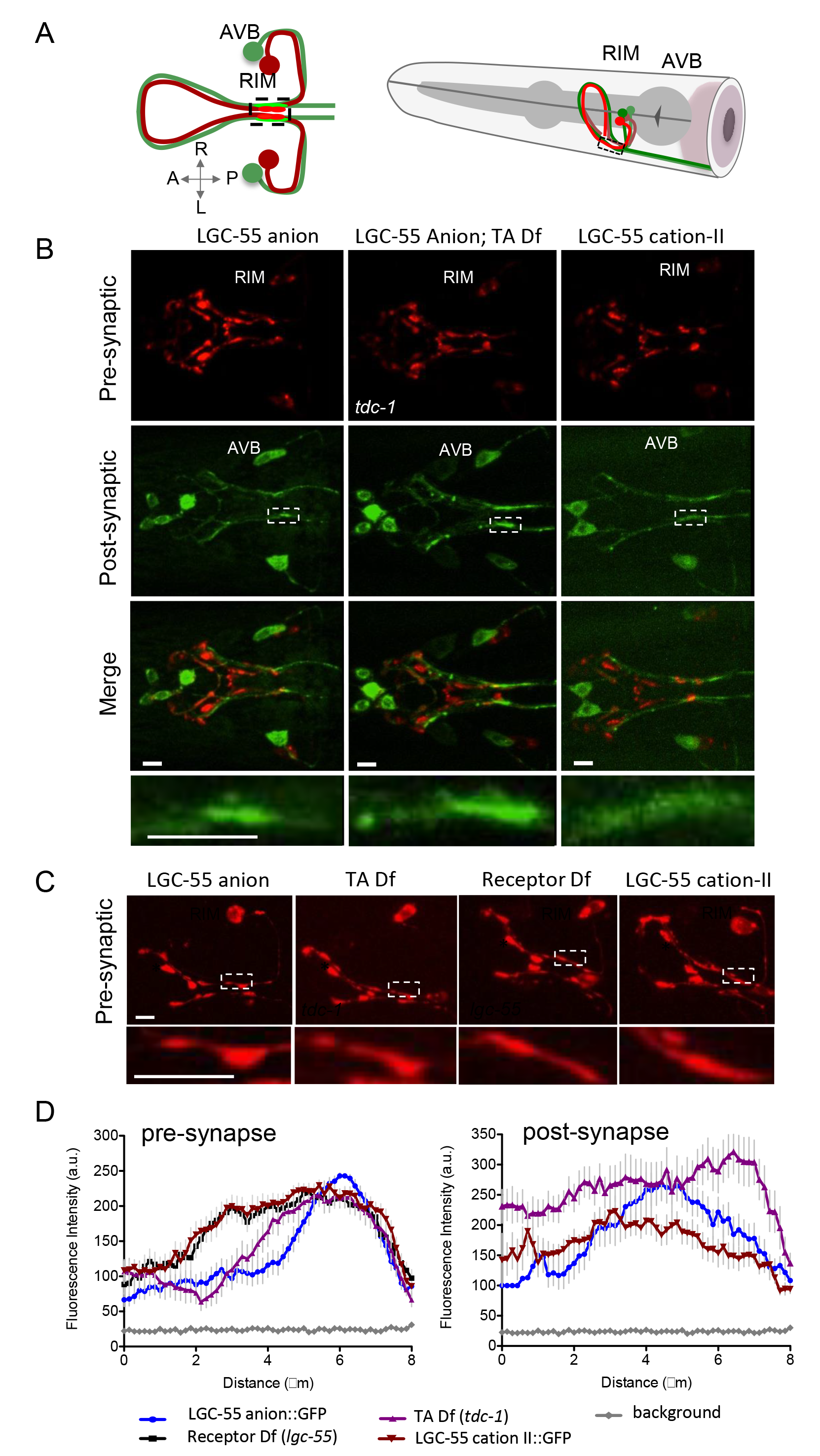

Supplement: S3 Fig — (A) Schematic diagram of the location of the synaptic outputs of the RIM onto the AVB (left: dorsal ventral view, right: side view of the head). (B) Representative images of the localization of GFP-tagged LGC-55 anion and LGC-55 cation-II opposite to presynaptic release sites from the RIM neuron (Pcex-1::RAB-3::mCherry) of Plgc-55 short::LGC-55 anion, TA-deficient (tdc-1), and Plgc-55 short::LGC-55 cation-II::GFP animals. The presynaptic marker area indicated by the rectangle is magnified below and correlates to connections with the RIM neuron. Scale bar is 3 um. (C) Representative images of synaptic vesicle marker RAB-3::mCherry in the RIM neuron of TA-deficient (tdc-1), receptor-deficient (lgc-55), LGC-55 anion, and LGC-55 cation-II transgenic animals. The area indicated by the rectangle is magnified below and corresponds to the area of synaptic outputs of the RIM with the AVB neuron. Scale bar, 3 um. (D) Fluorescence intensity of pre- and postsynaptic densities of the RIM-AVB synapse. Fluorescence intensity at the presynapse (left) and postsynapse (right) was measured in regular intervals over 8 μm in transgenic animals expressing mCherry::RAB-3 in the RIM of wild-type (Pcex-1::mCherry::RAB-3; Plgc-55::LGC-55::GFP, n = 19), TA-deficient (tdc-1(n3420); Pcex-1::mCherry::RAB-3; Plgc-55::LGC-55::GFP, n = 14), receptor-deficient (lgc-55(tm2913; Pcex-1::mCherry::RAB-3, n = 17), and cation-II (Pcex-1::mCherry::RAB-3; Plgc-55::LGC-55 cation-II::GFP, n = 11) animals. (TIF) [file pbio.1002238.s004.tif]

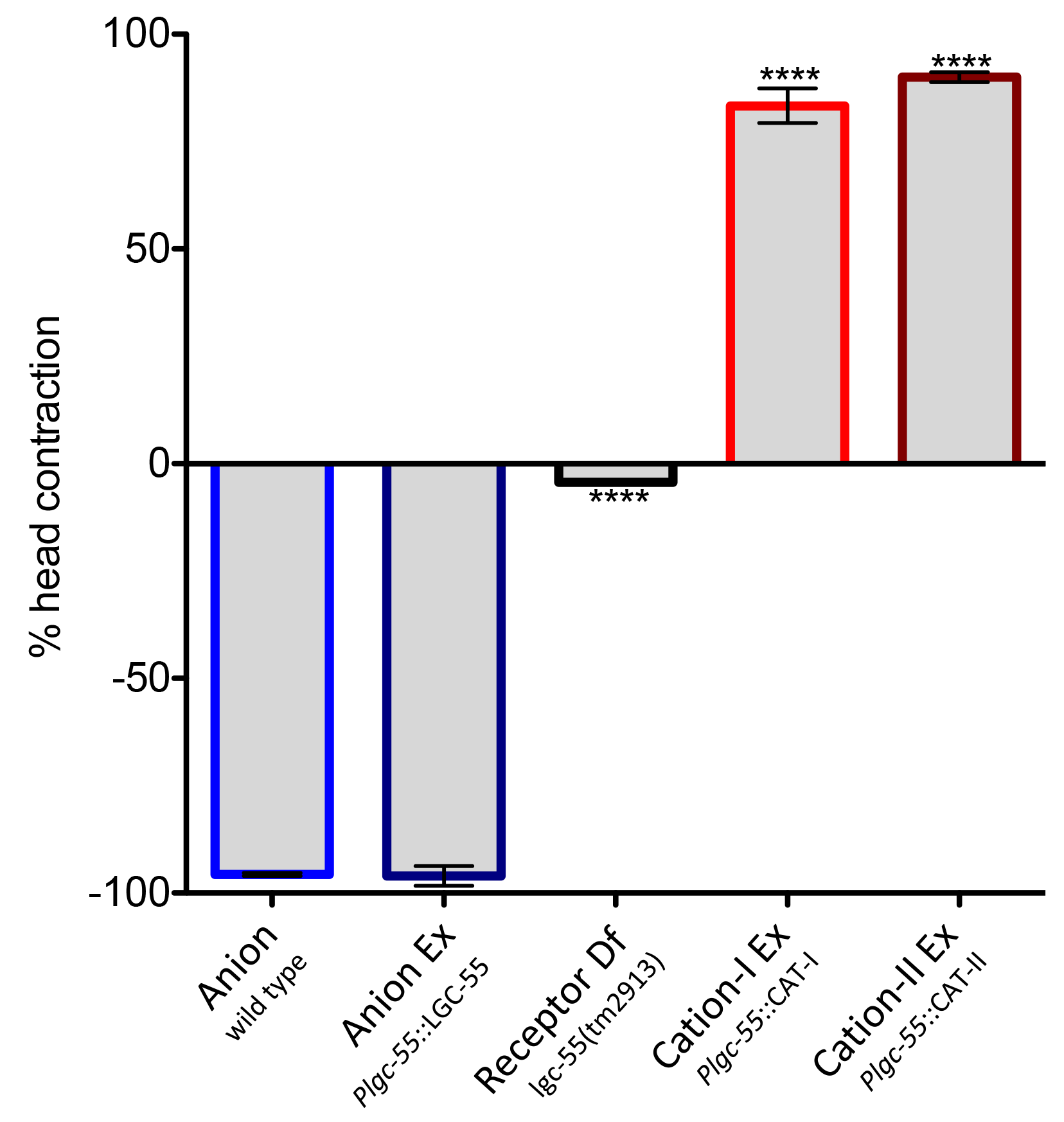

Supplement: S4 Fig — Shown is the percentage of animals that contract their necks in response to touch. Positive response indicates contraction, while negative response indicates relaxation. lgc-55 null mutants neither contract nor relax their necks, while transgenic animals expressing either LGC-55 anion or LGC-55 cation channels contract their necks in response to touch; n = 70 for all genotypes. See text for details. Statistical difference from LGC-55 anion, **** p ≤ 0.0001, two-tailed Student’s t test. (TIF) [file pbio.1002238.s005.tif]

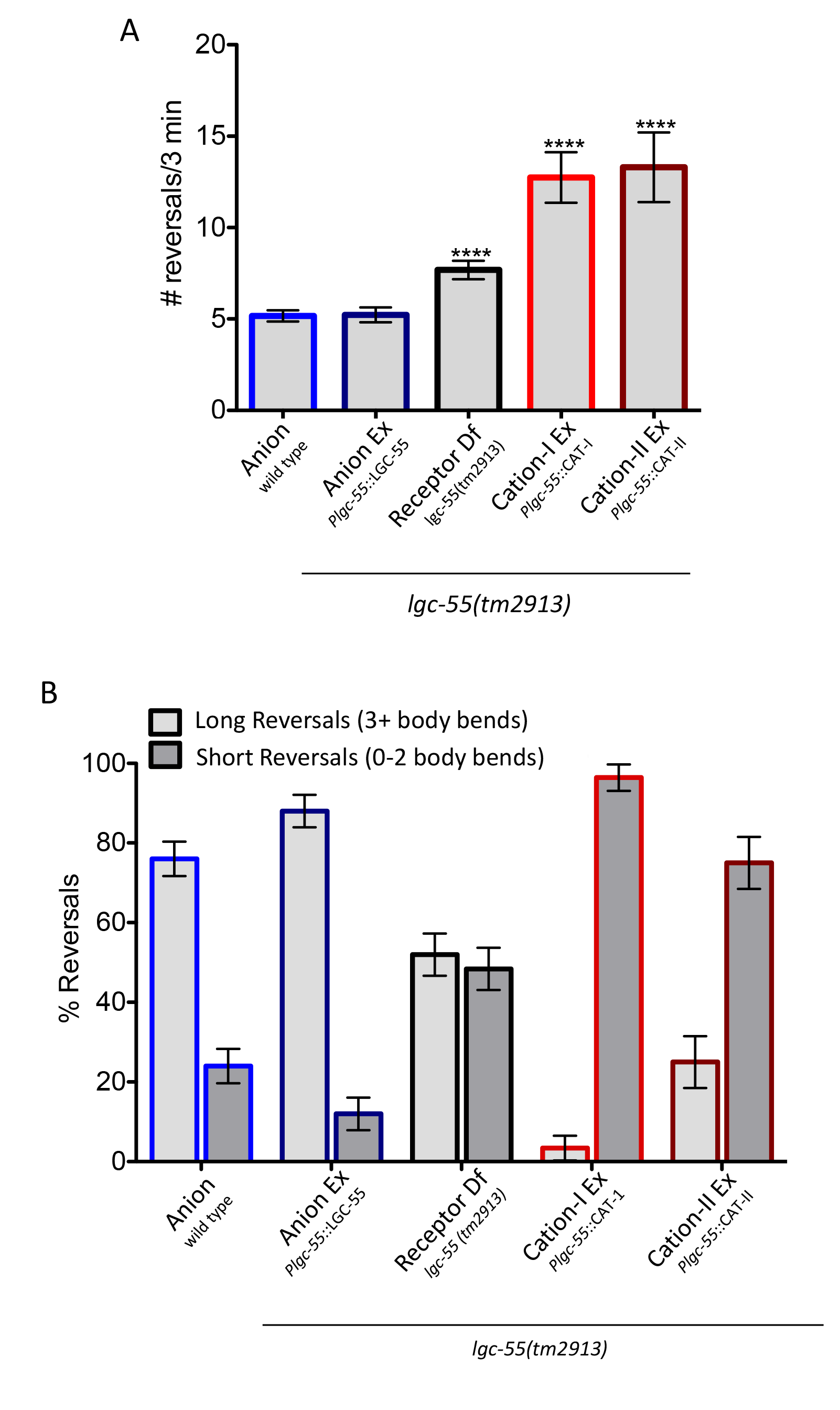

Supplement: S5 Fig — (A) Number of reversals made in 3 min of wild type, 5.2 ± 0.3 body bends, n = 30; LGC-55 rescue, 5.2 ± 0.4 body bends, n = 18; lgc-55(tm2913), 7.86 ± 0.5 body bends, n = 25; LGC-55 cation-I, 12.7 ± 1.4 body bends, n = 27; LGC-55 cation-II, 13.3 ± 1.9 body bends, n = 10. LGC-55 cation animals exhibit hyper reversal behavior. Statistical difference from LGC-55 anion. *** p < 0.0001, two-tailed Student’s t test. (B) Distribution of short (1–2 body bends) and long (3+ body bends) spontaneous reversals made in 3 min of wild type, n = 30; LGC-55 rescue, n = 18; lgc-55(tm2913), n = 25; LGC-55 cation-I, n = 27; LGC-55 cation-II, n = 10, p < 0.001, two-way ANOVA. LGC-55 is expressed in the AVB forward locomotion command neuron. In wild-type animals, spontaneous release of tyramine activates LGC-55 anion, causing a hyperpolarization of the AVB leading to a long reversal. In LGC-55 cation animals, spontaneous release of tyramine causes an activation of the AVB, leading to a shortened reversal length, and an increase in the number of short reversals made in 3 min. (TIF) [file pbio.1002238.s006.tif]

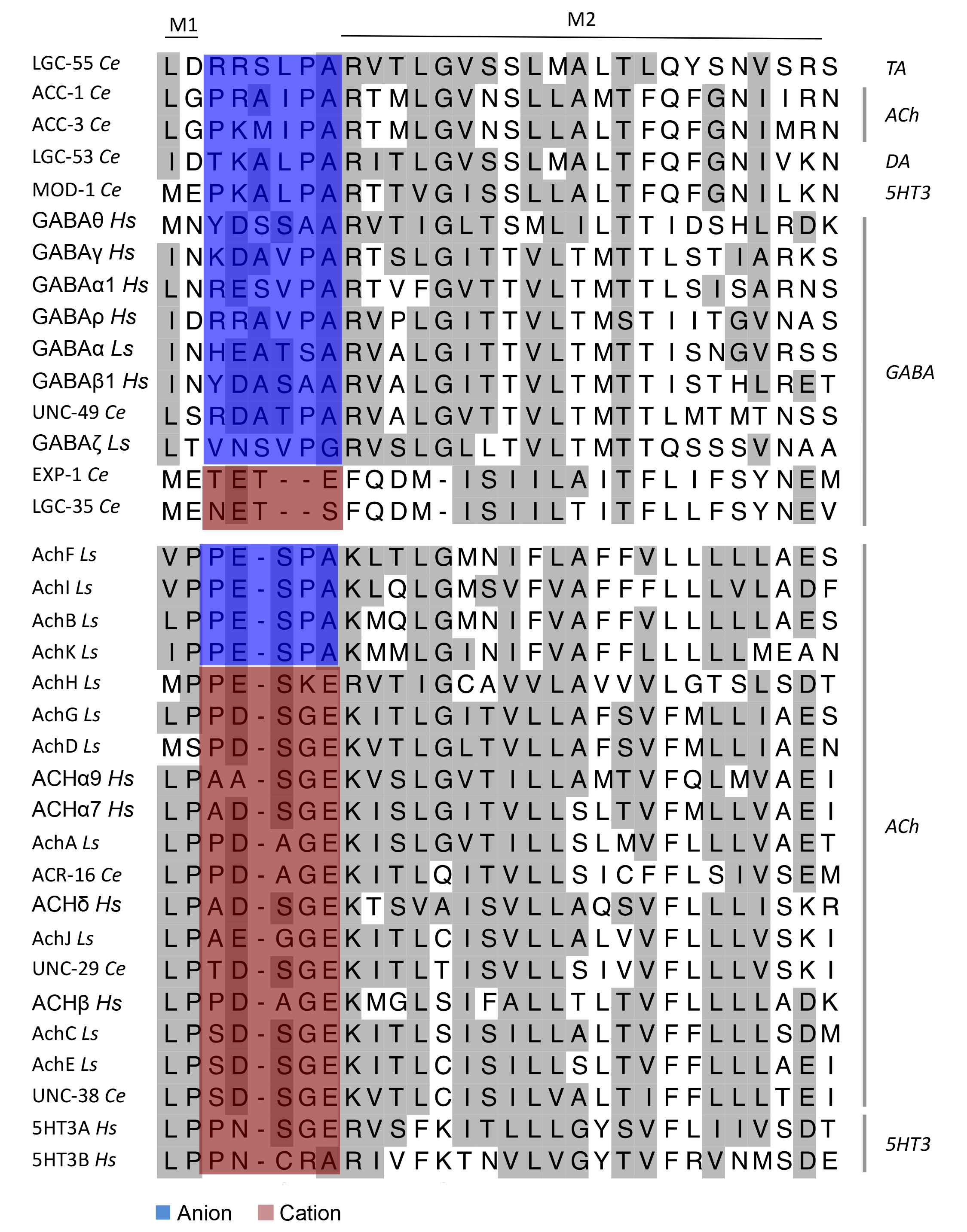

Supplement: S6 Fig — Shown is the alignment of the ion pore and M2 region of invertebrate and human LGICs used in the phylogenetic analysis in Fig 6. The neurotransmitters are indicated on the right. Identities are highlighted in grey, and blue shading indicates anionic channels, while red shading indicates cationic channels. Ce: C. elegans, Ls: Lymnaea stagnalis, Hs: Homo sapiens. Protein alignments were performed with ClustalW [40] and were carried out using MacVector Software (Accelrys). See Fig 7 for GenBank accession numbers. (TIF) [file pbio.1002238.s007.tif]
